# Gastric coinfection with thiopeptide-positive *Cutibacterium acnes* decreases FOXM1 and pro-inflammatory biomarker expression in a murine model of *Helicobacter pylori*-induced gastric cancer

**DOI:** 10.1128/spectrum.03450-23

**Published:** 2023-11-28

**Authors:** Courtney Lunger, Zeli Shen, Hilda Holcombe, Anthony J. Mannion, JoAnn Dzink-Fox, Susanna Kurnick, Yan Feng, Sureshkumar Muthupalani, Sebastian E. Carrasco, Keith T. Wilson, Richard M. Peek, M. Blanca Piazuelo, Douglas R. Morgan, Amanda L. Armijo, Melissa Mammoliti, Timothy C. Wang, James G. Fox

**Affiliations:** 1 Division of Comparative Medicine, Massachusetts Institute of Technology, Cambridge, Massachusetts, USA; 2 Division of Gastroenterology, Department of Medicine, Vanderbilt University Medical Center, Nashville, Tennessee, USA; 3 Division of Gastroenterology and Hepatology, Heersink School of Medicine, University of Alabama at Birmingham, Birmingham, Alabama, USA; 4 Division of Gastroenterology and Irvine Cancer Research Center, Columbia University, New York, New York, USA; University of Warwick, Coventry, United Kingdom

**Keywords:** *Helicobacter pylori*, non-*H. pylori* gastric bacteria, *Cutibacterium acnes*, thiopeptide, gnotobiotic mouse model, mouse model of *H. pylori*-induced gastric cancer, *Mus musculus*

## Abstract

*Helicobacter pylori* (*H. pylori*) causes a cascade from gastritis to gastric neoplasia. During the chronic stages, the oncogenic transcription factor forkhead box protein M1 (*FOXM1*) becomes increasingly expressed. Given certain strains of the gastric commensal *Cutibacterium acnes* (*C. acnes*) produce thiopeptides that directly inhibit FOXM1, we hypothesized that coinfection with thiopeptide-positive *C. acnes* would decrease *Foxm1* expression and alter *H. pylori*-induced pathogenesis. *C. acnes* was isolated from 31% of gastric biopsies from Nicaraguan patients, and thiopeptide-positive strains were identified by whole genome sequencing. Germ-free INS-GAS mice were inoculated with thiopeptide-positive *C. acnes*, *H. pylori* SS1, *H. pylori* 1 week before *C. acnes*, *C. acnes* 2 weeks before *H. pylori*, or no bacteria. At 17 weeks post-infection, males dosed with *C. acnes* followed by *H. pylori* exhibited increased inflammation scores by histopathology; however, males dosed with *H. pylori* followed by *C. acnes* exhibited reduced gastric *Foxm1*, pro-inflammatory cytokine (*Il-1β*, *Ifn-γ*, *Tnf-α*, *Il-17a*, and *iNOS*), regulatory cytokine (*Foxp3*) gene expression, pro-inflammatory IgG2a antibodies, and gastric lymph node RORγT expression compared to *H. pylori-*monoinfected mice. Male mice coinfected with *C. acnes* followed by *H. pylori* exhibited reduced gastric inflammatory markers (IL-17, IL-10, GM-CSF, M-CSF, MCP-1, MIP-1α, MIP-2, RANTES, and VEGF) by cytokine array analysis and reduced *H. pylori* gastric colonization. These data show that thiopeptide-positive *C. acnes* exhibited anti-inflammatory and anti-FOXM1 properties in the context of *H. pylori* gastritis.

**IMPORTANCE:**

*H. pylori* infects half of the world population and is the leading cause of gastric cancer. We previously demonstrated that gastric cancer risk is associated with gastric microbiota. Specifically, gastric urease-positive *Staphylococcus epidermidis* and *Streptococcus salivarius* had contrasting effects on *H. pylori*-associated gastric pathology and immune responses in germ-free INS-GAS mice. As gastritis progresses to gastric cancer, the oncogenic transcription factor *Foxm1* becomes increasingly expressed. In this study, we evaluated the gastric commensal *C. acnes*, certain strains of which produce thiopeptides that directly inhibit FOXM1. Thiopeptide-positive *C. acnes* was isolated from Nicaraguan patient gastric biopsies and inoculated into germ-free INS-GAS mice with *H. pylori*. We, therefore, asked whether coinfection with *C. acnes* expressing thiopeptide and *H. pylori* would decrease gastric *Foxm1* expression and pro-inflammatory cytokine mRNA and protein levels. Our study supports the growing literature that specific non-*H*. *pylori* gastric bacteria affect inflammatory and cancer biomarkers in *H. pylori* pathogenesis.

## INTRODUCTION

*Helicobacter pylori* (*H. pylori*) is the leading cause of gastritis and gastric neoplasia in humans. *H. pylori* has an infection rate of 50% worldwide according to the CDC and up to 80%–90% in developing countries (1 – 3). Following colonization of the human stomach by *H. pylori*, the Correa cascade describes a progressive cascade from normal gastric tissue to superficial gastritis, intestinal metaplasia, and eventually gastric cancer (4). During this *H. pylori*-associated cascade in humans, *H. pylori* produces microRNAs that progressively increase oncogenic transcription factor forkhead box protein M1 (FOXM1) expression (5 – 7). FOXM1 then facilitates gastric cancer cell migration and invasion (8 – 10). Elevation in FOXM1 is a poor prognostic factor and mediates resistance to docetaxel, a chemotherapeutic (11, 12). FOXM1 has, therefore, been identified as a potential therapeutic target for gastric cancer. In C57BL/6J mice infected with *H. pylori* SS1, FOXM1 was elevated at a single time point of 8 months post-infection (5).

Previous studies in our laboratory have explored the role of non-*H*. *pylori* gastric bacteria in *H. pylori* pathogenesis and gastric cancer risk (13, 14). *Staphylococcus epidermidis* and *Streptococcus salivarius* isolated from patient gastric tissues had contrasting effects on *H. pylori*-induced pathogenesis in germ-free (GF) INS-GAS mice (15). At 5 months post-infection, mice coinfected with *Streptococcus salivarius* had higher histopathologic scores, and mice coinfected with *Staphylococcus epidermidis* had lower pro-inflammatory cytokine gene expression, compared to *H. pylori* monoinfection.

*Cutibacterium acnes* (*C. acnes*) is a commensal bacterium that colonizes multiple tissues, including skin, oral cavity, respiratory tract, and gastrointestinal tract (16 – 18). A novel thiopeptide from *C. acnes* was recently discovered. Thiopeptides are microbially produced peptides with antibacterial, immunomodulatory, and anticancer properties (19, 20). Prototype thiopeptides include thiostrepton from *Streptomyces* species, siomycin A from *Streptomyces sioyaensis*, and berninamycin from *Streptomyces bernensis*. The novel thiopeptide, named cutimycin, has a nearly identical structure to berninamycin A (18). Thiopeptides inhibit bacterial protein synthesis, with activity against Gram-positive and Gram-negative bacteria (19, 20). The anticancer action of thiopeptides is attributed to direct inhibition of FOXM1 and blockade of the proteasome (19, 20). Due to its instability in acidic environments, thiostrepton is not bioavailable when administered orally (21) but is FDA approved in a topical antibiotic formulation for veterinary use (Animax Ointment, Dechra Pharmaceuticals, Northwich, United Kingdom).

Thiopeptide-positive strains of *C. acnes* inhibited *Staphylococcus aureus* growth, supporting an antimicrobial role for *C. acnes* thiopeptide similar to previously evaluated thiopeptides (18). Thiopeptide-positive *C. acnes* also downregulated expression of the oncogenic transcription factor FOXM1 and inhibited cell proliferation in human primary prostate epithelial cells (22). Thus, thiopeptides and their bacterial producers, including *C. acnes*, have pharmacologic potential against infection and cancer, which we sought to explore in the context of *H. pylori*-induced gastric cancer. This study aims to characterize the effect of *C. acnes*, specifically a thiopeptide-positive strain, on *H. pylori* pathogenesis and continues our previous work elucidating the effect of non-*H*. *pylori* gastric bacteria on *H. pylori* pathogenesis. Given the potential ability for *C. acnes* thiopeptide to directly inhibit the oncogenic transcription factor FOXM1, which becomes progressively increased in human *H. pylori* infection, we hypothesized that coinfection of *H. pylori* and thiopeptide-positive *C. acnes* would alter inflammatory biomarkers and decrease FOXM1 expression in GF INS-GAS mice.

## RESULTS

### Gastric *Foxm1* progressively increases in *H. pylori*-infected mice, paralleling *H. pylori* infection in humans

**Fig 1:**
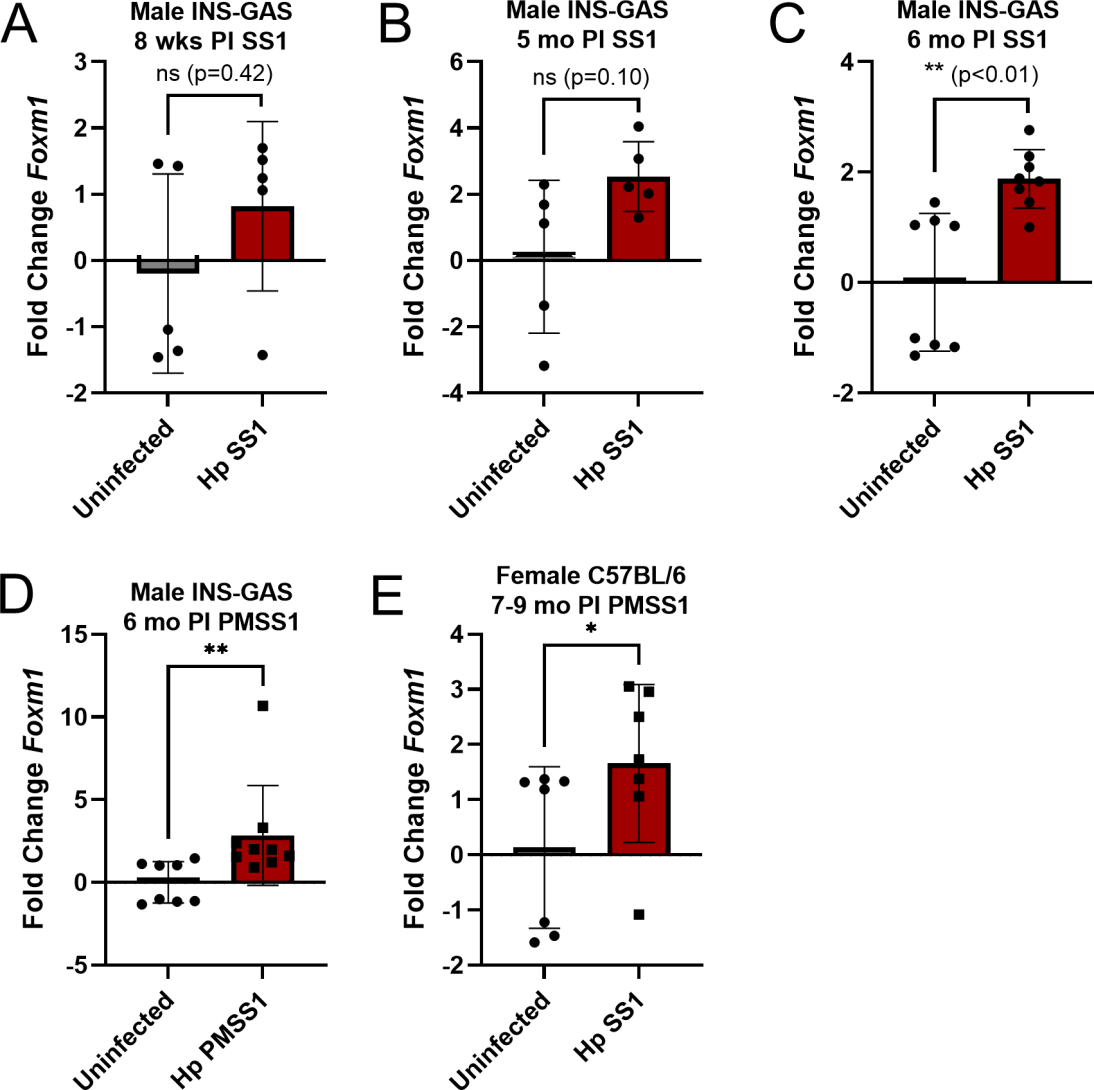
Retrospective *Foxm1* expression. mRNA quantified by quantitative PCR using gastric tissue samples from previous studies. Gastric *Foxm1* in INS-GAS male mice at (**A**) 8 weeks, (**B**) 5 months, and (**C**) 6 months post-infection with *H. pylori* SS1. (**D**) Gastric *Foxm1* in INS-GAS male mice at 6 months post-infection with *H. pylori* PMSS1. (**E**) Gastric *Foxm1* in C57BL/6 female mice at 7–9 months PI with *H. pylori* SS1. Gastric *Foxm1* was progressively increased in male INS-GAS mice infected with *H. pylori* SS1 and was similarly increased with *H. pylori* PMSS1 infection and in females C57BL/6 mice. Hp, *H. pylori*. *P < 0.05, **P < 0.01.

To demonstrate that *Foxm1* is elevated in *H. pylori*-infected mice in parallel to what occurs in humans, *Foxm1* gene expression was quantified in gastric tissue samples from our previous studies (14 – 16). Gastric *Foxm1* was not elevated in INS-GAS male mice at 8 weeks or 5 months post-infection with *H. pylori* SS1 (Fig. 1A and B) but was elevated by 6 months post-infection (Fig. 1C). Gastric *Foxm1* was also elevated at 6 months post-infection with *H. pylori* PMSS1, as well as in a different mouse strain and sex, C57BL/6 females, infected with *H. pylori* SS1 at 7–9 months post-infection (Fig. 1D and E).

### Bacterial culture of gastric biopsy samples from Nicaraguan patients

Bacterial isolation was performed on 36 human gastric samples from Nicaragua. Bacteria cultured from these samples represented 22 genera, with the top five most frequently isolated genera being *Streptococcus* (*n* = 25), *Rothia* (*n* = 19), *Actinomyces* (*n* = 15), *Cutibacterium* (*n* = 11), and *Veillonella* (*n* = 7) (Table S1).

*C. acnes* was cultured from 11 gastric biopsies out of 36 (31%), which were from seven female patients and four male patients (Table 1). Most of these biopsies were positive for *H. pylori* by histopathology (*n* = 10/11) (Table 1), while only a subset was positive for *H. pylori* by culture (*n* = 3/11) (Table S2). Most samples had a histopathologic diagnosis of non-atrophic gastritis (NAG) (*n* = 7/11); the remaining showed gastric intestinal metaplasia (GIM) (*n* = 4/11) (Table 1).

**TABLE 1:** *C. acnes* isolated from Nicaraguan gastric samples^*a*^

*C. acnes* strain ID	Sex	*H. pylori* status	Histopathology	Thiopeptide PCR	WGS performed?
18-1849-A2	F	+	GIM	−	No
18-1851-A4	F	+	NAG	+	Yes
18-1857-A1	F	−	NAG	−	Yes
18-1859-A1	F	+	GIM	−	No
18-1863-A1	F	+	NAG	−	No
18-1864-C6	M	+	GIM	−	No
18-1869-C3b	F	+	NAG	+	Yes
18-1871-A4	M	+	NAG	−	No
18-1873-C1	M	+	NAG	−	No
18-1879-A3	F	+	NAG	+	Yes
18-1881-C2	M	+	GIM	−	No

^*a*^ Eleven strains of *C. acnes* were isolated from human antral biopsy samples. Shown for each gastric biopsy are the MIT accession number, clone ID, sex of the patient, *H. pylori* status, histopathologic diagnosis, results of thiopeptide PCR, and whether whole genome sequencing was performed. NAG, non-atrophic gastritis; GIM, gastric intestinal metaplasia.

### Whole genome sequencing reveals thiopeptide biosynthetic gene cluster in *C. acnes* isolated from human stomachs

**Fig 2:**
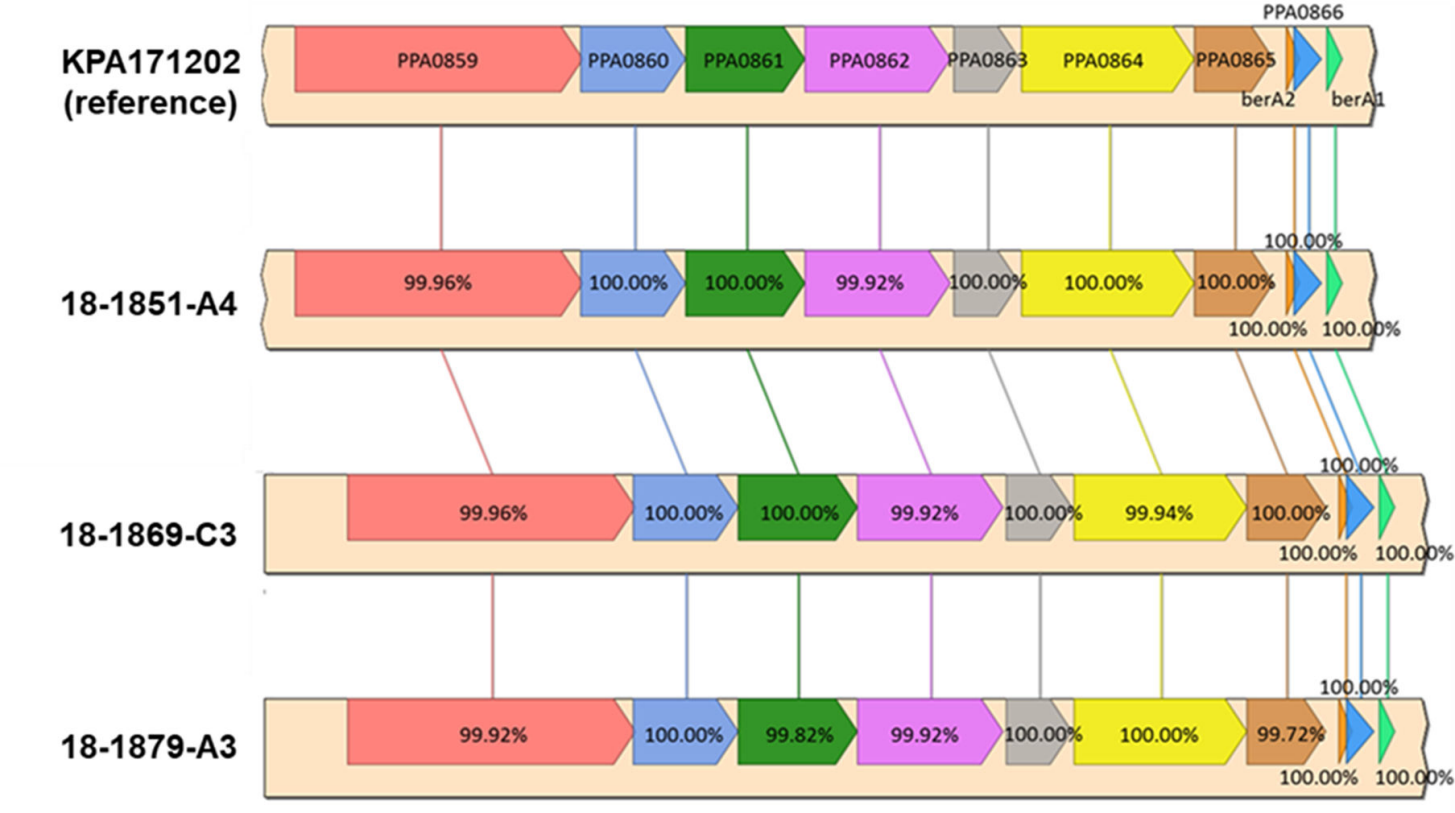
Sequencing of thiopeptide biosynthetic gene cluster. *C. acnes* MIT strains 18-1879-A3, 18-1869-C3, and 18-1851-A4 strains isolated from human gastric tissue have complete thiopeptide biosynthetic gene clusters by whole genome sequencing with greater than 99% identity compared to the reference *C. acnes* strain.

Whole genome sequencing of four *C. acnes* MIT isolates revealed that three were thiopeptide positive. All thiopeptide-positive strains were from gastric biopsies diagnosed with NAG. BLAST analysis and syntenic alignment confirmed that these *C. acnes* strains, named MIT 18-1851-A4, MIT 18-1869-C3, and MIT 18-1879-A3, had complete thiopeptide biosynthesis gene clusters with >99% identity compared to the reference strain KPA171202 (Fig. 2). None of the genes in the thiopeptide biosynthesis cluster were found in the genome of the fourth *C. acnes* strain, named MIT 18-1857-A1. Thiopeptide-encoding *C. acnes* MIT 18-1851-A4, MIT 18-1869-C3, and MIT 18-1879-A3 had larger genomes and more protein gene annotations compared to MIT 18-1857-A1 (thiopeptide negative) (Table 2).

**TABLE 2:** Whole genome sequencing of *C. acnes* strains^*a*^

*C. acnes* strain ID	Contigs	Size	%GC content	Protein coding sequences (CDS)	RNA genes	GenBank
18-1857-A1	10	2,483,440	60.1	2,447	48	WOWG00000000
18-1851-A4	17	2,547,745	60.0	2,502	48	WOWH00000000
18-1869-C3	14	2,547,366	60.0	2,492	48	WOWI00000000
18-1879-A3	12	2,545,723	60.0	2,483	47	WOWJ00000000
*C. acnes* KPA171202	1	2,560,265	60.0	2,422	48	AE017283

^*a*^ Genome summary statistics for four *C. acnes* MIT isolates and reference strain KPA171202.

### Thiopeptide-positive *C. acnes* inhibited the growth of *H. pylori in vitro*

**Fig 3:**
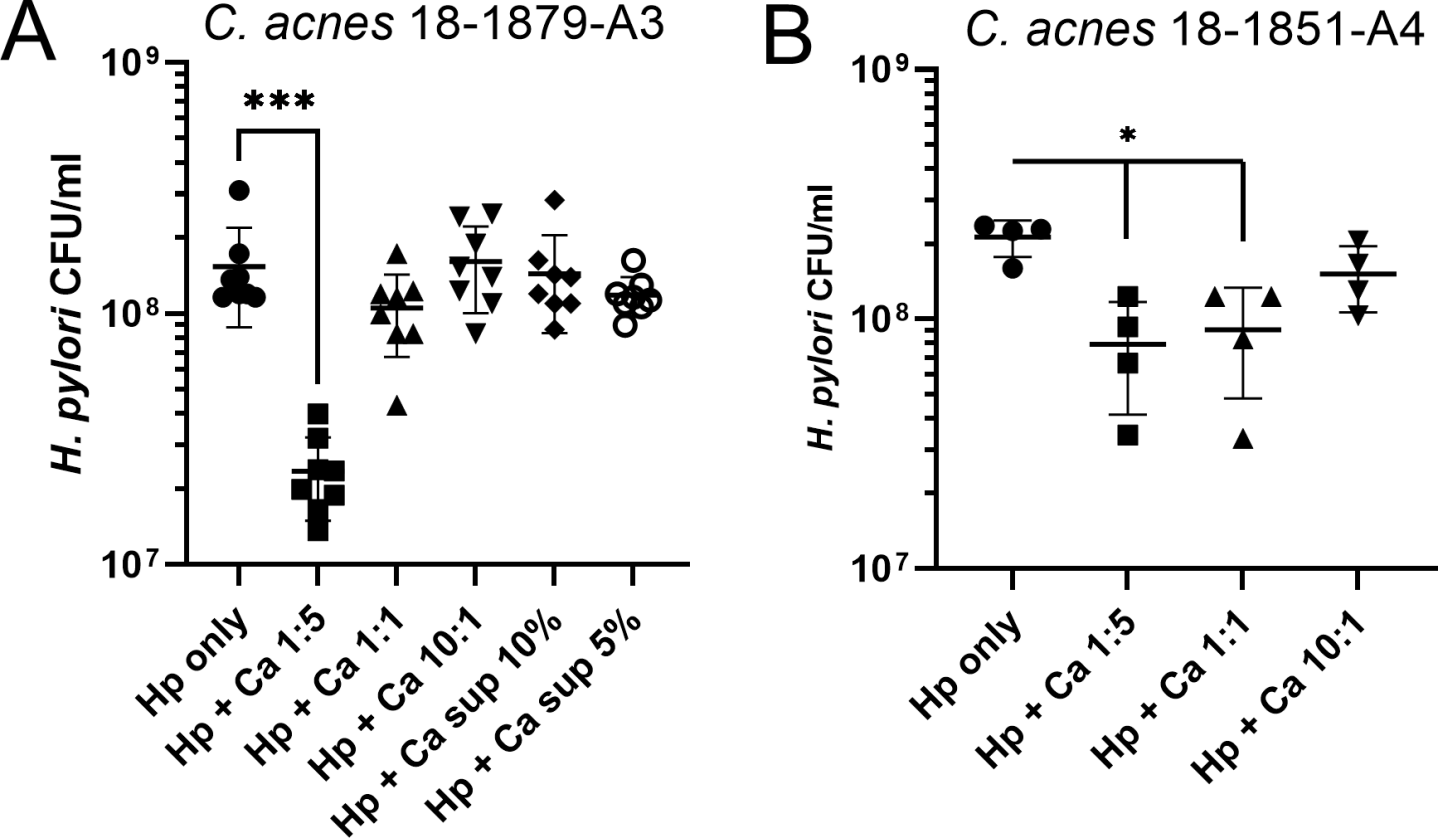
Thiopeptide-positive *C. acnes* inhibition assay. *H. pylori* PMSS1 (0.2 OD _600_ ) was grown in monoculture or with thiopeptide-positive *C. acnes* MIT 18-1879-A3 (**A**) and MIT 18-1851-A4 (**B**) at MOI 1:5, 1:1, or 10:1. *H. pylori* PMSS1 was also incubated with 5% or 10% supernatant from *C. acnes* monoculture. CFU/mL were quantified following 72-hour incubation. Both thiopeptide-positive strains of *C. acnes* inhibited the growth of *H. pylori in vitro*. Hp, *H. pylori* PMSS1; Ca, *C. acnes;* sup, supernatant. *P < 0.05, ***P < 0.001.

Given the known antimicrobial effects of thiopeptides, two of these thiopeptide-positive strains of *C. acnes* were tested for antibacterial activity against *H. pylori*. When cocultured with thiopeptide-positive *C. acnes* MIT 18-1879-A3 or MIT 18-1851-A4, *H. pylori* growth was reduced at a multiplicity of infection (MOI) of 1:5 for *C. acnes* MIT 18-1879-A3 and MOI of 1:5 or 1:1 for *C. acnes* MIT 18-1851-A4 (Fig. 3). Bacterial supernatant at concentrations of 5% or 10% from thiopeptide-positive *C. acnes* MIT 18-1879-A3 culture did not inhibit the growth of *H. pylori* (Fig. 3A).

### Thiopeptides inhibit gastric cancer cell line growth and reduce *Foxm1* expression

**Fig 4:**
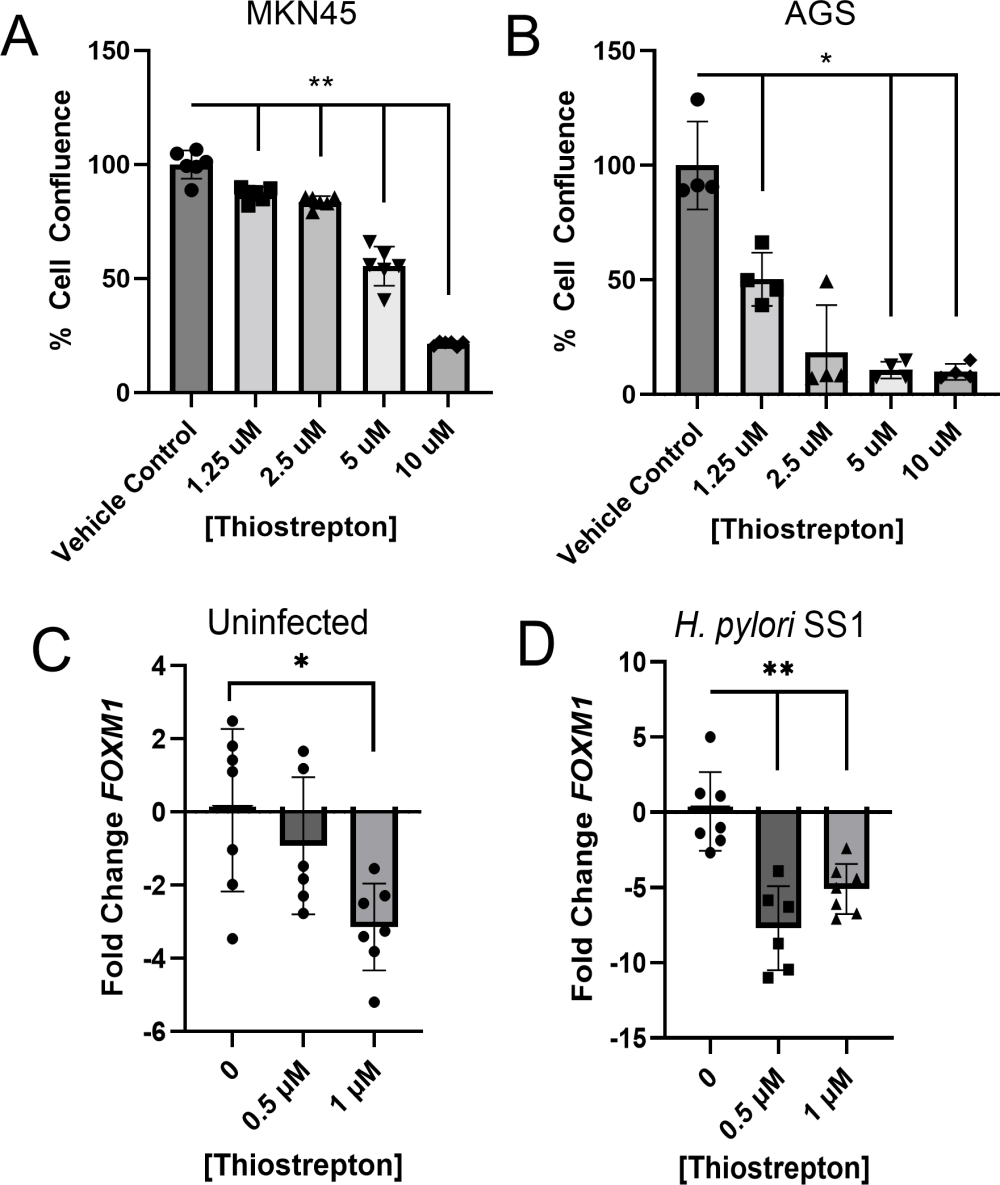
Effect of thiostrepton on gastric cancer cell growth and *Foxm1* expression. Percent epithelial cell confluence of (**A**) MKN45 and (**B**) AGS gastric cancer cell lines after 72 hours following incubation with vehicle control or escalating concentrations of thiostrepton. Percent cell confluence was measured using the MTT assay. AGS gastric cancer cells incubated with thiostrepton at 0, 0.5, and 1 µM and incubated (**C**) with *H. pylori* strain SS1 or (**D**) uninfected at an MOI of 1:100 or vehicle control (DMSO). Following incubation at 37°C for 24 hours, fold changes of *Foxm1* compared to the housekeeping gene *Gapdh* were determined by qPCR. Thiostrepton inhibited the proliferation of gastric cancer cell lines in a dose-dependent manner and decreased *Foxm1* expression in *H. pylori*-infected and uninfected AGS cells. *P < 0.05, **P < 0.01.

The direct effect of thiopeptides on cancer cell growth and *Foxm1* expression was evaluated using thiostrepton, the thiopeptide produced by *Streptomyces* spp., as *C. acnes* thiopeptide is not commercially available. Thiostrepton inhibited MKN45 and AGS gastric cancer cell line proliferation *in vitro* in a dose-dependent manner (Fig. 4). AGS cells, both with and without concurrent *H. pylori* infection, exhibited decreased *Foxm1* gene expression at 24 hours after exposure to thiostrepton 0.5 or 1 µM (Fig. 4).

### Male mice coinfected with *C. acnes* prior to *H. pylori* exhibited decreased *H. pylori* gastric colonization

**Fig 5:**
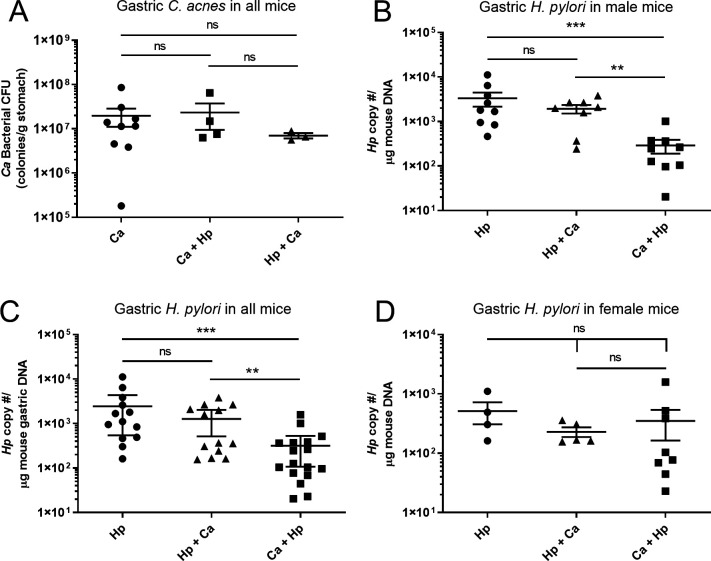
*C. acnes* and *H. pylori* colonization in germ-free mice. (**A**) *C. acnes* CFU/mL in *C. acnes* or coinfected mice 17 weeks post-infection. *H. pylori* copy number per microgram of DNA in gastric tissue by qPCR from (**B**) male mice, (**C**) mice of both sexes, and (**D**) female mice infected with *H. pylori* alone or coinfected with *C. acnes*. Hp, *H. pylori* SS1 strain; Ca, *C. acnes*; Hp + Ca, mice infected with *H. pylori* followed by *C. acnes*; Ca + Hp, mice dosed with *C. acnes* prior to *H. pylori*. **P < 0.01, ***P < 0.001.

To evaluate the effect of thiopeptide-positive *C. acnes* on *H. pylori*-induced gastritis *in vivo*, GF INS-GAS mice were inoculated with thiopeptide-positive *C. acnes* MIT 18-1879-A3, *H. pylori* SS1, *H. pylori* 1 week before *C. acnes* (Hp + Ca), *C. acnes* 2 weeks before *H. pylori* (Ca + Hp), or no bacteria. Necropsies were performed at 17 weeks post-infection, and gastric colonization of *H. pylori* and *C. acnes* was quantified. *C. acnes* was isolated from the oral cavity, stomach, and feces of all tested *C. acnes*-colonized mice. *C. acnes* colonization did not differ between groups dosed with *C. acnes* alone or *C. acnes* with *H. pylori* (Fig. 5A). Male mice infected with *C. acnes* prior to *H. pylori* had decreased gastric *H. pylori* colonization compared to *H. pylori* alone (Fig. 5B and C). *H. pylori* colonization did not differ between male mice infected with *H. pylori* alone and coinfected with *H. pylori* followed by *C. acnes* or between *H. pylori*-monoinfected and coinfected females (Fig. 5B through D).

### Coinfected male mice exhibited increased histopathologic inflammatory scores

**Fig 6:**
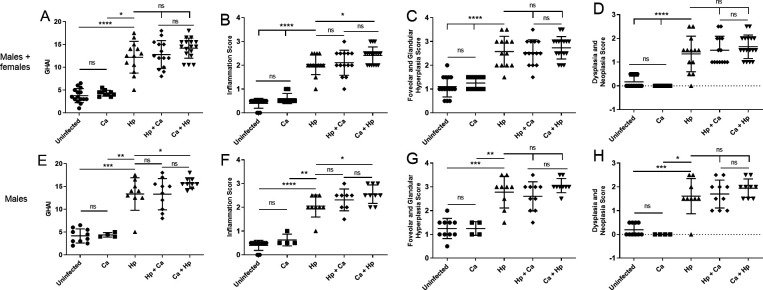
Gastric histopathology. Combined gastric histologic activity index (GHAI) as well as inflammation, foveolar and glandular hyperplasia, and dysplasia and neoplasia scores in both sexes (**A–D**) and males (**E–H**) 17 weeks post-infection. Gastric inflammation scores were increased in male mice colonized with *C. acnes* prior to *H. pylori*. Hp, *H. pylori* SS1 strain; Ca, *C. acnes*; Hp + Ca, mice infected with *H. pylori* followed by *C. acnes*; Ca + Hp, mice dosed with *C. acnes* prior to *H. pylori*. *P < 0.05, **P < 0.01, ***P < 0.001, ****P < 0.0001.

At 17 weeks post-infection with *H. pylori*, gastric tissue from all groups was examined histologically. Histopathologic indices were increased in *H. pylori*-infected animals compared to uninfected and *C. acnes* controls (Fig. 6A through H). Gastric histologic activity indices (GHAI) did not differ between animals of both sexes in coinfection groups compared to *H. pylori* alone (Fig. 6A); however, male mice colonized with *C. acnes* followed by *H. pylori* had increased GHAI due to increased inflammation scores (Fig. 6E and F). Total histopathology, inflammation, and foveolar or glandular hyperplasia scores did not differ between coinfected and monoinfected females (Fig. S1A through C). Dysplasia and neoplasia scores were increased in females dosed with *C. acnes* prior to *H. pylori* compared to *H. pylori* alone (Fig. S1D).

### Decreased inflammatory biomarkers and *Foxm1* in the stomach of coinfected mice

**Fig 7:**
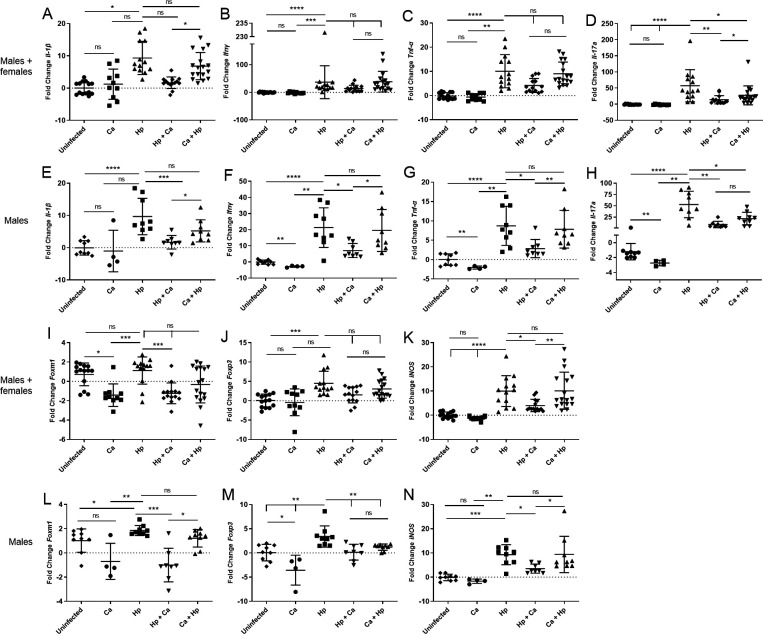
Gastric cytokine and *Foxm1* gene expression. mRNA levels of *Il-1β*, *Ifn-γ*, *Tnf-α*, *Il-17a*, *Foxm1*, *Foxp3*, and *iNOS* in male and female mice (**A–D and I–K**) and male mice (**E–H and L–N**) 17 weeks post-infection. Gastric pro-inflammatory cytokine and *Foxm1* expression was decreased in male mice infected with *H. pylori* followed by *C. acnes* compared to *H. pylori* monoinfection. Hp, *H. pylori* SS1 strain; Ca, *C. acnes*; Hp + Ca, mice infected with *H. pylori* followed by *C. acnes*; Ca + Hp, mice dosed with *C. acnes* prior to *H. pylori*. *P < 0.05, **P < 0.01, ***P < 0.001, ****P < 0.0001.

The changes in gastric inflammatory biomarker and oncogene *Foxm1* expression in response to *H. pylori* infection were measured by qPCR. Pro-inflammatory cytokine expression was increased in gastric tissue samples from *H. pylori*-infected animals compared to uninfected and *C. acnes* controls, especially for male mice (Fig. 7A through K; Fig. S2A through J). *C. acnes*-colonized male mice exhibited decreased Th1-associated cytokines (*Ifn-γ* and *Tnf-α*), Th17-associated cytokine (*Il-17a*), Treg-associated *Foxp3*, and *Foxm1* compared to uninfected mice (Fig. 7F, G, H, I, and M). *Foxm1* was increased in *H. pylori*-infected males compared to uninfected (Fig. 7L). Males infected with *H. pylori* and then *C. acnes* exhibited reduced pro-inflammatory gastric cytokines (*Il-1β*, *Ifn-γ*, *Tnf-α*, *Il-17a*, and *iNOS*), regulatory cytokines (*Foxp3*), and *Foxm1* (Fig. 7D, E, F, G, H, I, K, L, and M) compared to monoinfection. Females infected with *H. pylori* and then *C. acnes* exhibited decreased *Il-1β* expression (Fig. S2B). Males infected with *C. acnes* followed by *H. pylori* had decreased *Il-17a* and *Foxp3* compared to *H. pylori* alone (Fig. 7H and M).

**Fig 8:**
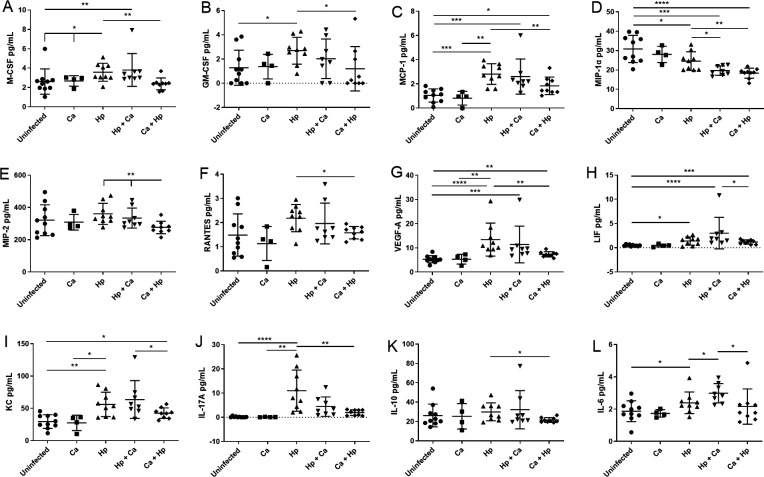
Gastric inflammatory protein levels. (**A**) M-CSF, (**B**) GM-CSF, (**C**) MCP-1, (**D**) MIP-1α, (**E**) MIP-2, (**F**) RANTES, (**G**) VEGF-A, (**H**) LIF, (**I**) KC, (**J**) IL-17A, (**K**) IL-10, and (**L**) IL-6 in male germ-free INS-GAS mice 17 weeks post-infection. Pro-inflammatory gastric protein levels were decreased in male mice colonized with *C. acnes* prior to *H. pylori*. Hp, *H. pylori* SS1 strain; Ca, *C. acnes*; Hp + Ca, mice infected with *H. pylori* followed by *C. acnes*; Ca + Hp, mice dosed with *C. acnes* prior to *H. pylori*. *P < 0.05; **P < 0.01, ***P < 0.001, ****P < 0.0001.

Given the decrease in mRNA expression of gastric inflammatory markers observed in coinfected male mice, protein levels of gastric cytokines were also quantified in male mice by cytokine array. IFN-γ, eotaxin, G-CSF, IP-10, and MIG were increased in *H. pylori*-infected mice compared to uninfected and *C. acnes*-colonized controls (Fig. S3A through F). Male mice colonized with *C. acnes* followed by *H. pylori* showed decreased macrophage pro-inflammatory factors M-CSF, GM-CSF, MCP-1, MIP-1α, and MIP-2 (Fig. 8A through E), pro-inflammatory marker IL-17a (Fig. 8J), T-reg-associated IL-10 (Fig. 8K), and platelet-associated cytokines RANTES and VEGF (Fig. 8F and G) compared to *H. pylori* monoinfection. Gastric IL-6 was increased in male mice infected with *H. pylori* followed by *C. acnes* (Fig. 8L). Gastric levels of IL-1β, IL-2, IL-3, IL-4, IL-5, IL-7, IL-9, IL-12p40, IL-12p70, IL-13, IL-15, LIX, MIP-1β, and TNFα did not differ between groups of male mice (data not shown).

**Fig 9:**
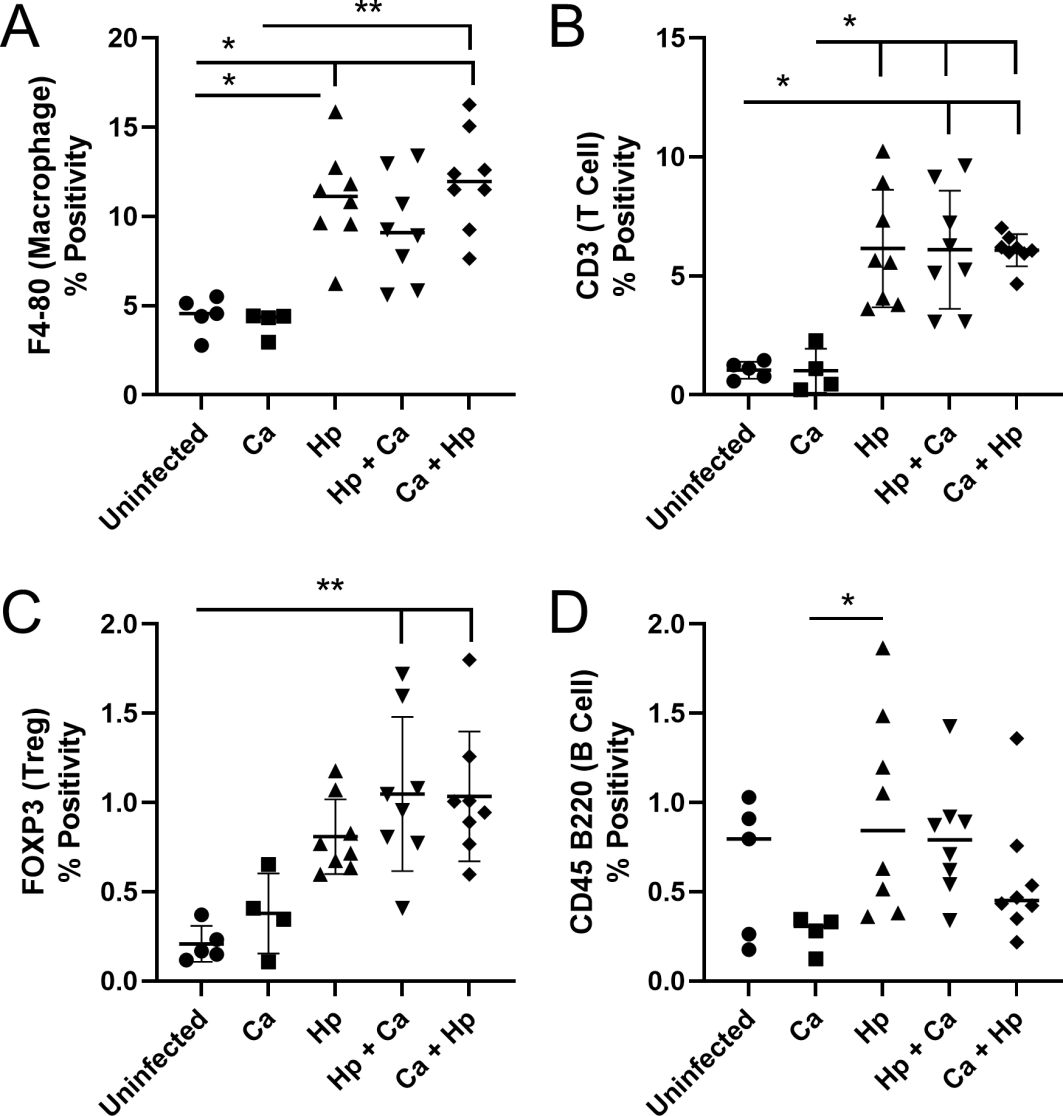
Gastric IHC. IHC staining of (**A**) F4/80 for macrophages, (**B**) CD3 for T cells, (**C**) FOXP3 for Treg cells, and (**D**) CD45 B220 for B cells in the gastric tissue of germ-free INS-GAS mice that were uninfected, colonized by *C. acnes*, infected with *H. pylori*, infected with *H. pylori* prior to *C. acnes*, or dosed with *C. acnes* prior to *H. pylori* at 17 weeks post-infection. Quantification performed for A–D. Gastric inflammatory cells were increased in *H*. pylori-infected mice compared to uninfected and *C. acnes* controls. Hp, *H. pylori* SS1 strain; Ca, *C. acnes*; Hp + Ca, mice infected with *H. pylori* followed by *C. acnes*; Ca + Hp, mice dosed with *C. acnes* prior to *H. pylori*. *P < 0.05, **P < 0.01.

Due to the decreased mRNA expression and protein levels of gastric Th1, Th17, and Treg-associated cytokines in the stomach between coinfected and *H. pylori*-monoinfected male mice, inflammatory cell subsets were quantified in gastric tissue by immunohistochemistry (IHC). F4/80+ macrophages, CD3+ T cells, FOXP3+ Treg cells, and CD45 B220+ B cells were increased in the gastric mucosa of animals infected with *H. pylori* compared to uninfected and *C. acnes* controls (Fig. 9; Fig. S6). Low numbers of MPO+ neutrophils were present in all groups (Fig. S6). There were no differences between groups by qualitative assessment of inflammatory cells. When comparing *H. pylori* monoinfection and coinfection groups, gastric immune cell numbers did not differ.

### Coinfected males had exhibited less inflammatory serum antibody and draining lymph node responses to *H. pylori* infection

**Fig 10:**
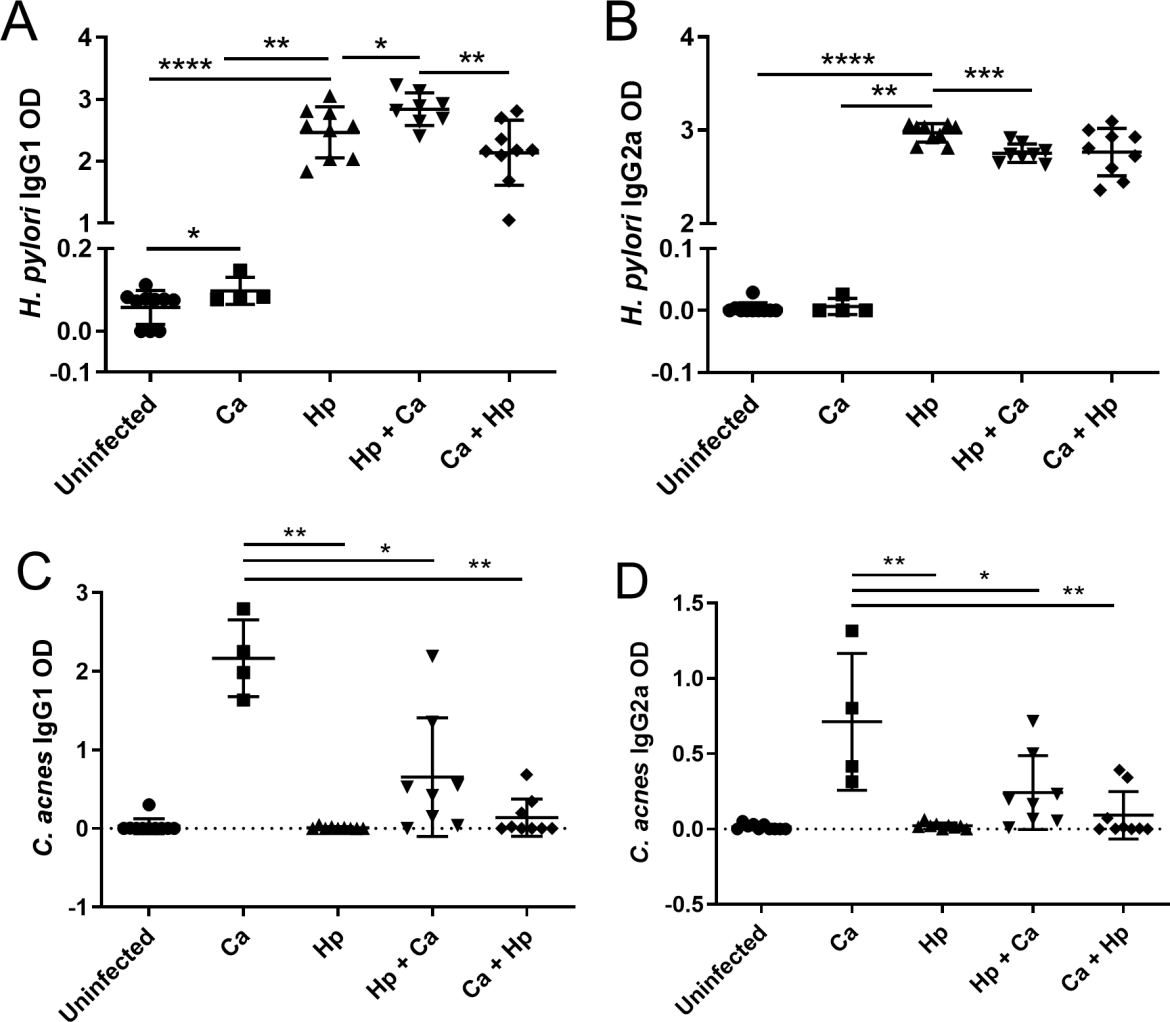
Serum antibody response to *H. pylori* and *C. acnes*. Anti-*H*. *pylori* and *C. acnes* serum anti-inflammatory IgG1 (**A and C**) and pro-inflammatory IgG2a (**B and D**) antibodies measured by ELISA in male germ-free INS-GAS mice that were uninfected, colonized by *C. acnes*, infected with *H. pylori*, infected with *H. pylori* prior to *C. acnes*, or dosed with *C. acnes* prior to *H. pylori* 17 weeks post-infection. Anti-inflammatory IgG1 antibodies were increased and pro-inflammatory IgG2a antibodies were decreased in male mice infected with *H. pylori* prior to *C. acnes* compared to *H. pylori* Monoinfection. Hp, *H. pylori* SS1 strain; Ca, *C. acnes*; Hp + Ca, mice infected with *H. pylori* followed by *C. acnes*; Ca + Hp, mice dosed with *C. acnes* prior to *H. pylori*. *P < 0.05, **P < 0.01, ***P < 0.001, ****P < 0.0001.

Based on the anti-inflammatory effect of coinfection on gastric cytokine responses in male mice, the serum antibody and draining lymph node T cell responses to *H. pylori* infection were also evaluated. In males infected with *H. pylori* prior to *C. acnes*, anti-inflammatory IgG1 *H. pylori* antibodies were increased, and pro-inflammatory IgG2a *H. pylori* antibodies were decreased compared to *H. pylori* alone (Fig. 10A and B). Coinfected females had decreased anti-inflammatory IgG1 *H. pylori* antibodies compared to *H. pylori* alone, but no difference in pro-inflammatory IgG2a antibodies between *H. pylori*-infected groups (Fig. S4A and B). Anti-*C*. *acnes* IgG1 and IgG2a antibodies were decreased in coinfected males compared to *C. acnes* alone (Fig. 10C and D). Coinfected females showed no differences in anti-*C*. *acnes* IgG1 and IgG2a antibodies compared to *C. acnes* alone (Fig. S4C and D).

**Fig 11:**
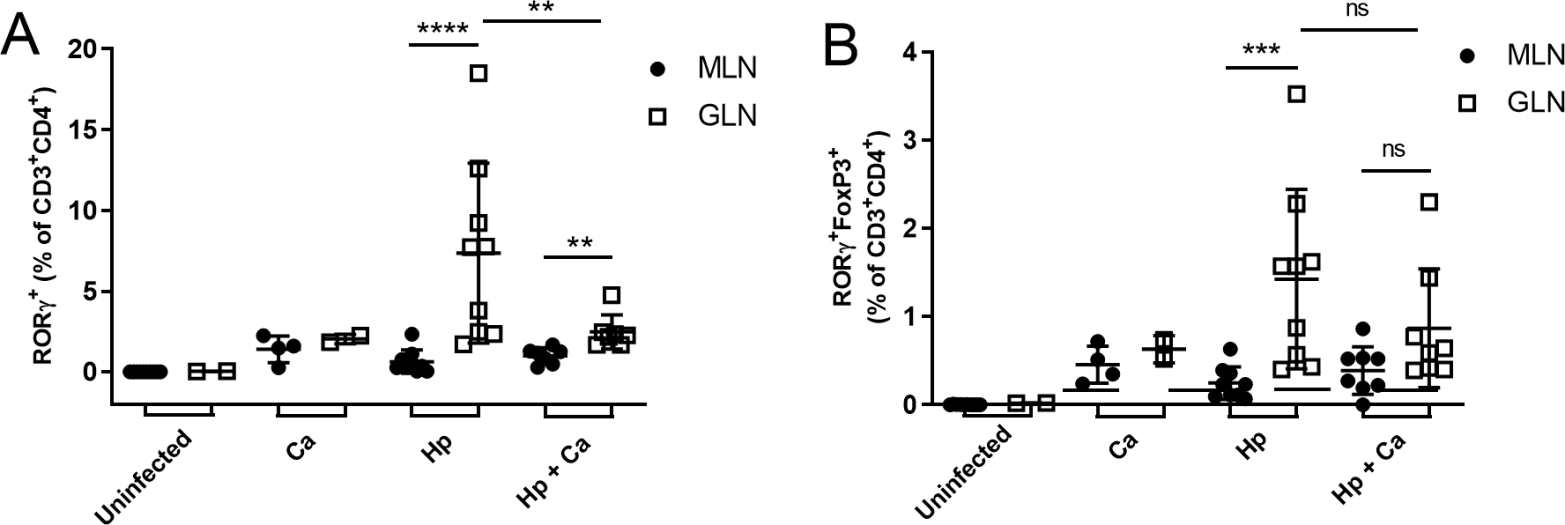
Flow cytometry of mesenteric and gastric lymph nodes. (**A**) Percent RORγT expression in CD4+ T cells in gastric and mesenteric lymph nodes. (**B**) Percent FOXP3 RORγT double-positive CD4+ T cells in gastric and mesenteric lymph nodes. Percent RORγT expression was decreased in gastric lymph nodes of mice coinfected with *H. pylori* followed by *C. acnes* compared to *H. pylori* monoinfection at 17 weeks post-infection. Percent RORγT and FOXP3 RORγT double-positive cells were increased in gastric lymph nodes from mesenteric for *H. pylori-infected* mice. Hp, *H. pylori* SS1 strain; Ca, *C. acnes*; Hp + Ca, mice infected with *H. pylori* followed by *C. acnes*; Ca + Hp, mice dosed with *C. acnes* prior to *H. pylori*. *P < 0.05, **P < 0.01, ***P < 0.001, ****P < 0.0001.

In the draining gastric lymph nodes (GLN), there were fewer RORγT+ Th17 cells for *H. pylori* and *C. acnes-*coinfected males compared to *H. pylori* alone (Fig. 11A). In *H. pylori*-infected animals, RORγT+ Th17 cells and RORγT+ FOXP3+ double-positive Treg cells were increased in GLNs compared to mesenteric lymph nodes (MLNs) (Fig. 11A and B). *C. acnes*- and/or *H. pylori*-colonized mice had an increased percentage of T-Bet+ Th1 cells and decreased percentage of FOXP3+ Treg cells compared to uninfected controls (Fig. S5). Percentage of T-Bet+ Th1 cells and FOXP3+ Treg cells in GLNs and MLNs did not differ between coinfected and monoinfected mice (Fig. S5).

## DISCUSSION

FOXM1 is a pro-oncogenic transcription factor overexpressed in many cancers, including *H. pylori*-induced gastric cancer (5 – 7). To ascertain whether gene expression of the oncogenic transcription factor *Foxm1* is elevated in *H. pylori*-infected mice, we measured *Foxm1* in mice previously studied by our laboratory (23 – 25). *H. pylori* infection of both sexes (INS-GAS males and C57BL/6 females) increased *Foxm1* gene expression compared to uninfected controls. This was true for *H. pylori* PMSS1 and SS1, the latter of which is deficient in the bacterial type IV secretion system needed to deliver the oncogenic protein CagA to host epithelial cells, which suggests the mechanism of *Foxm1* elevation does not depend on this phenomenon. *H. pylori* SS1 was chosen for the *in vivo* studies as PMSS1 results in varied colonization levels and duration and the loss of *Cag* expression during infection (26). Thus, mouse models accurately recapitulated the progressive increase in gastric *FOXM1* seen in *H. pylori*-infected humans.

We then evaluated the prototype thiopeptide, thiostrepton, for anticancer properties, finding that thiostrepton decreased *FOXM1* expression in AGS cells and inhibited the growth of MKN-45 and AGS gastric cancer epithelial cell lines. The decrease in *FOXM1* expression may have been a direct effect of thiostrepton or caused by cellular growth inhibition. Cytotoxicity was less likely the cause of decreased gastric cancer cell growth because thiostrepton at the same concentrations decreased breast cancer cell growth without affecting normal breast epithelial cells (27). In addition, the FDA-approved Animax Ointment contains 2,500 units/mL of thiostrepton per the Safety Data Sheet, and thiostrepton has an activity of 900 units/mg per the USP reference standard with a molecular weight of 1,664.9 g/mol; thus, the thiostrepton concentration is 1.7 × 10^3^ µM. This is approximately 1,000× the concentration used in our study and suggests that tumor suppression was not the result of thiostrepton toxicity.

In Nicaraguan gastric biopsies, 31% (11 of 36) were positive for *C. acnes*; by contrast, 5% (2 of 37) of gastric biopsies from our previously reported Colombian population were positive for *C. acnes* (15). Other bacterial isolates from Nicaraguan samples included *Streptococcus* spp., *Actinomyces* spp., and *Prevotella* spp., which are often associated with oral and respiratory microbiota. Whole genome sequencing found three of four *C. acnes* strains to be thiopeptide positive. PCR for the thiopeptide biosynthetic gene cluster aligned with whole genome sequencing results (Table 1). Interestingly, the three thiopeptide-positive biopsies had a diagnosis of non-atrophic gastritis, while five of nine thiopeptide-negative biopsies demonstrated the more severe histopathologic diagnosis of gastric intestinal metaplasia. This may suggest that thiopeptide-positive *C. acnes* is associated with less severe gastric pathology than thiopeptide-negative *C. acnes*, but further investigation is warranted.

The effect of thiopeptide-positive strains of *C. acnes* on the growth of *H. pylori* was tested *in vitro* using two positive strains isolated by our laboratory; both inhibited the growth of *H. pylori in vitro* in a dose-dependent manner, potentially due to direct effects or thiopeptide secretion. Thiopeptide-positive *C. acnes* 18-1879-A3 was inoculated into GF INS-GAS mice along with *H. pylori* SS1. Importantly, gastric *Foxm1* expression was decreased in mice coinfected with *H. pylori* prior to *C. acnes* compared to *H. pylori* monoinfection. In GF INS-GAS mice infected with *H. pylori*, coinfection with *C. acnes* inhibited *Foxm1* elevation, which is a key step in the progression of gastritis to gastric cancer. Future studies may compare *Foxm1* expression and gastric cancer rates in *H. pylori-*colonized patients with and without concurrent thiopeptide-positive *C. acnes* colonization.

Consistent with previous studies, *H. pylori* infection of GF INS-GAS mice increased GHAI, which included inflammation, epithelial defects, foveolar and glandular hyperplasia, and dysplasia and neoplasia scores (15). An increase in the epithelial defect scores was seen in females dosed with *C. acnes*, but overall histopathology scores were not affected. Total histopathologic scores did not differ in male and female coinfected mice compared to *H. pylori* monoinfection. However, male mice colonized by *C. acnes* followed by *H. pylori* exhibited increased inflammation scores compared to *H. pylori* monoinfection, which translated to an increase in total histopathologic scores. This may be due to GF mice having more prominent immune responses to commensal bacteria than mice whose immune systems have previously been primed by the presence of microbiota. In fact, higher gastric histopathology scores were noted in *H. pylori*-infected GF INS-GAS mice colonized with Altered Schaedler’s Flora (*Clostridium* species ASF356, *Bacteroides* species ASF519, and *Lactobacillus murinus* ASF361) compared to *H. pylori* monocolonization (28). Decreased anti-*C*. *acnes* serum antibodies in coinfected mice compared to *C. acnes* alone also support there being immunomodulatory effects in coinfection. In addition, dosing mice with *C. acnes* prior to *H. pylori* decreased *H. pylori* colonization of the stomach in males. It is possible that increased inflammation in mice dosed with *C. acnes* prior to *H. pylori* led to selective clearing of *H. pylori*. However, macrophage, T and B lymphocyte, and neutrophil numbers did not differ between *H. pylori* monoinfection and coinfection by quantitative analysis of digital IHC images.

Gastric gene expression was quantified for Th1 (*Il-1β*, *Ifn-γ*, *Tnf-α*, and *iNOS*), Th17 (*Il-17a* and *Il-22*), and Treg (*Foxp3* and *Il-10*) cytokines, which are increased in *H. pylori* infection (29 – 32). *C. acnes* alone had an anti-inflammatory effect in the stomach of GF INS-GAS mice with decreased mRNA expression of *Ifn-γ, Tnf-α*, *Il-17a*, and *Foxp3* compared to uninfected mice. Consistent with previous studies, *Il-1β*, *Ifn-γ*, *Tnf-α*, *Il-17a*, and *Foxp3* mRNA levels were increased in *H. pylori-*infected animals compared to uninfected controls (15). Coinfected male mice infected with *H. pylori* prior to *C. acnes* infection had decreased gastric mRNA expression of *Il-1β*, *Ifn-γ*, *Tnf-α*, *Il-17a*, *iNOS*, and *Foxp3* compared to *H. pylori* monoinfection. Coinfected males dosed with *C. acnes* followed by *H. pylori* exhibited a decrease in both mRNA and protein expression of IL-17a, as well as mRNA expression of *Foxp3*, compared to *H. pylori* monoinfection. Female mice infected with *H. pylori* followed by *C. acnes* exhibited decreased gastric mRNA expression of *Il-1β*, but no other cytokines, compared to *H. pylori*-monoinfected female mice. As previously demonstrated in INS-GAS male mice and similar to human males, INS-GAS male mice showed more robust effects from *H. pylori* infection than females (33, 34).

Gastric inflammatory protein levels were quantified in male mice, including macrophage-associated proteins given histiocytic activation occurs in *H. pylori* infection (32, 35, 36). Gastric protein levels of GM-CSF and M-CSF, which regulate macrophage differentiation, MCP-1, a key monocyte chemoattractant, and MIP-1α and MIP-2, inflammatory proteins produced by macrophages, were decreased in male mice coinfected with *C. acnes* followed by *H. pylori* compared to *H. pylori* monoinfection. RANTES, a chemokine driving infiltration of inflammatory immune cells, and VEGF, which increases vascularity during inflammation, were also decreased in this coinfection group. Interestingly, gastric IL-6 was increased in mice infected with *H. pylori* followed by *C. acnes* compared to *H. pylori* monoinfection. While IL-6 is commonly regarded as a pro-inflammatory cytokine that is increased in *H. pylori* gastritis and gastric cancer (29, 37), the cytokine also has anti-inflammatory effects, including promoting alternative activation of macrophages to an anti-inflammatory phenotype (38). Overall, male coinfected mice had decreased gastric pro-inflammatory cytokine and protein expression. Interestingly, mice infected with *H. pylori* followed by *C. acnes* showed more effects on mRNA expression, while coinfection with *C. acnes* followed by *H. pylori* showed more effects on protein levels. The order in which bacteria are introduced appears to have an effect on *H. pylori* inflammatory biomarkers, potentially due to differences in the inflammatory state of the stomach when *H. pylori* is introduced.

The anti-inflammatory effect of *C. acnes* coinfection was also seen in the anti-*H*. *pylori* serum antibody response and Th17 cell differentiation in gastric lymph nodes. Coinfected male mice dosed with *H. pylori* prior to *C. acnes* infection had an increase in anti-inflammatory, Th2-associated IgG1 and a decrease in pro-inflammatory, Th1-associated IgG2a compared to *H. pylori* monoinfection. Mice in this coinfection group also had a decrease in RORγT expression in CD4+ T cells in gastric lymph nodes, suggesting that the anti-inflammatory effect of *C. acnes* extended to draining gastric lymph nodes.

Immune cell populations were also compared between gastric and mesenteric lymph nodes. The gastric lymph node, as the major draining lymph node of the stomach, showed increases in pro-inflammatory Th1 (T-BET+) and Th17 (RORγT+) cells, as well as decreases in Treg (FOXP3+) cells, with *H. pylori* infection. This is consistent with human gastric lymph nodes showing increased RORγT in CD4+ T cells in the early stages of gastric cancer (31). This study found elevated FOXP3 expression, while our study found decreased FOXP3 expression, which may reflect the inflammatory and regulatory balance of the immune system at the time of tissue collection. In addition, a recently described CD4+ T cell, which is double positive for Th17-associated transcription factor RORγT and Treg-associated transcription factor FoxP3, has been found in the gastrointestinal tract and exhibits enhanced regulatory function (39, 40). However, to the authors’ knowledge, this cell type has not been characterized in the context of *H. pylori* infection. We show that in *H. pylori* infection, RORγT FOXP3 double-positive CD4+ T cells were increased in the gastric lymph node compared to mesenteric lymph node in GF mice. These data support the evaluation of gastric lymph node in *H. pylori* infection, as there may be a more robust immune response than in mesenteric lymph nodes.

Further studies are needed to evaluate the effect of *C. acnes* on *H. pylori* in the timeframe of gastric cancer development, which occurs around 7 months post-infection in GF INS-GAS males (41). In Korean, Japanese, and Chinese populations, *C. acnes* was abundant in gastric cancer tissues; however, the thiopeptide status was not reported in these studies, and it is unclear whether *C. acnes* acts as a bystander or an active contributor in gastric cancer development (42 – 44).

In conclusion, this study demonstrated that thiopeptide-positive *C. acnes* has anti-inflammatory, antimicrobial, and anti-FOXM1 effects *in vitro* and in a GF mouse model of *H. pylori* gastritis. Comparison with a thiopeptide-negative strain of *C. acnes* will be needed to determine whether the overall anti-inflammatory effects of *C. acnes* may be due to thiopeptide inhibition, other bacterial secreted factors, or direct effects of the bacteria. Given we recently reported that *Staphylococcus epidermidis* and *Streptococcus salivarius* in GF INS-GAS mice have contrasting effects on *H. pylori* gastritis, this study adds to the body of literature supporting a role for non-*H*. *pylori* gastric bacteria in immunomodulating the progression of *H. pylori*-induced gastritis and gastric cancer.

## MATERIALS AND METHODS

### Gastric *Foxm1* expression in *H. pylori-*infected mice

Gastric tissues were obtained from previous studies (23 – 25), including male INS-GAS mice 8 weeks (*n* = 5 uninfected, 5 infected), 5 months (*n* = 5 uninfected, 5 infected), and 6 months (*n* = 8 uninfected, 8 infected) post-infection with *H. pylori* SS1, male INS-GAS mice (*n* = 8 uninfected, 9 infected) 6 months post-infection with *H. pylori* PMSS1, and female C57BL/6 mice (*n* = 7 uninfected, 7 infected) 6 months post-infection with *H. pylori* PMSS1. RNA was extracted using TRIzol reagent (Thermo Fisher Scientific, Waltham, MA) and converted to cDNA using a high-capacity cDNA Archive kit following the manufacturer protocol (Thermo Fisher Scientific, Waltham, MA). cDNA levels for *Foxm1* mRNA were measured by quantitative PCR using commercial primers and probes and compared to the housekeeping gene *Gapdh*.

### Bacterial isolations and patient population

Thirty-six human antral biopsy specimens were collected from Nicaraguan patients. Participation was voluntary, and informed consent was obtained. The ethics committees of participating Nicaraguan hospitals and the Institutional Review Board of Vanderbilt University approved all study protocols. Subjects included 14 males and 22 females between 22 and 69 years old. Histopathologic diagnosis was non-atrophic gastritis in 20 biopsies, multifocal atrophic gastritis in two biopsies, and gastric intestinal metaplasia in 14 biopsies. Specimens were frozen at −80°C in thioglycolate, then thawed in an anaerobic atmosphere, and homogenized. For aerobic culture, homogenates were plated onto chocolate agar, blood agar, MacConkey agar, and Brucella broth medium containing 10% fetal calf serum. The plates were incubated at 37°C in 5% CO_2_ for 24–48 hours. For anaerobic culture, homogenates were plated onto pre-reduced Brucella blood agar plates and inoculated into thioglycolate broth. The cultures were incubated at 37°C in an anaerobic chamber (Coy Lab Products, Jackson County, Michigan) with mixed gas (10% CO_2_, 10% H_2_, 80% N_2_) for 48 hours. For microaerobic culture to detect the growth of *H. pylori*, homogenates were plated onto *H. pylori*-selective plates (45) and Brucella blood agar plates after passing through a 0.65-mm syringe filter. The plates were placed into a vented jar filled with mixed gas (10% CO_2_, 10% H_2_, 80% N_2_) and incubated at 37°C for up to 3 weeks. Isolated bacterial strains were identified by 16S rRNA sequencing.

### Characterization of *C. acnes* strains by whole genome sequencing

DNA was isolated from *C. acnes* strains MIT 18-1857-A1, MIT 18-1851-A4, MIT 18-1869-C3, and MIT 18-1879-A3 using the Roche High Pure PCR product purification kit after prior incubation with 50 µL of lysozyme (50 mg/mL) and 10 µL of mutanolysin (2,500 units/mL) for 30 minutes at 37°C. Barcoded libraries were constructed using the QIAseq FX DNA library kit and sequenced with an Illumina MiSeq instrument (2 × 300 bp reads). Raw sequence reads were decontaminated of adapters and quality trimmed using Trim Galore (version: 0.6.1) and Cutadapt (version: 2.2) from the FastqUtils tool hosted by PATRIC (46). SPAdes (version: 3.10.0) was used for *de novo* contig assembly followed by genome annotation by Rapid Annotations using Subsystems Technology, both hosted by PATRIC (46). Average nucleotide identities (ANI) were calculated with JSpeciesWS (47). ANI values ≥95% were considered the same species. The syntenic alignment of the thiopeptide gene island between genomes was determined with SimpleSynteny (48) using *C. acnes* reference strain KPA171202.

### *In vitro* assay for the effect of *C. acnes* on *H. pylori* growth

*H. pylori* PMSS1 was grown on 5% sheep blood agar plates under microaerobic conditions of 80% N_2_:10% H_2_:10% CO_2_ for 48 hours. Bacteria were collected into Brucella broth with 10% FBS. *C. acnes* MIT 18-1879-A3 and MIT 18-1851-A4 were cultured on 5% sheep blood agar plates for 24 hours under the same conditions as *H. pylori. H. pylori* (0.2 OD/mL) was cocultured with *C. acnes* at MOI 1:5, 1:1, or 10:1 OD_600_/mL. *C. acnes* MIT 18-1879-A3 was also grown in liquid culture of 10% fetal bovine serum in brain heart infusion (BHI) broth overnight. Bacterial supernatant was prepared by centrifuging the liquid culture at 12,000 rpm for 10 minutes, then passing through a 0.22-µm filter. Bacterial supernatant from *C. acnes* MIT 18-1879-A3 was added to *H. pylori* culture at concentrations of 5% or 10%. Mixtures were incubated in microaerobic conditions for 24 hours, after which 3 µL of serial dilutions was placed onto blood agar plates containing vancomycin (20 µg/mL), bacitracin (200 µg/mL), and nalidixic acid (20 µg/mL). *H. pylori* colonies were counted after 4 days of incubation.

### The effect of thiostrepton on gastric cancer cell lines

MKN45 and AGS gastric cancer epithelial cell lines were cultured in Dulbecco’s Modified Eagle’s Medium (ATCC, Manassas, VA) containing 10% fetal calf serum (Sigma-Aldrich, St. Louis, MO) and 1% antibiotic-antimycotic (100 units/mL penicillin, 100 µg/mL streptomycin, and 0.25 µg/mL amphotericin B; Gibco/Thermo Fisher Scientific, Grand Island, NY) at 37°C with 5% CO_2_. MKN45 cells and AGS cells were exposed to vehicle control or 1.25, 2.5, 5, or 10 µM thiostrepton. The MTT assay for cell proliferation was performed as described previously (49).

The direct effect of thiostrepton on 12 human *H. pylori* strains was tested at a concentration of 1.0 µM of thiostrepton, and no inhibitory effect was observed (data not shown). Therefore, concentrations of thiostrepton of 1.0 µM or less were utilized to evaluate the effect of thiostrepton on uninfected and *H. pylori*-infected AGS gastric cancer cells. AGS gastric cancer cells were incubated with thiostrepton at 0, 0.5, or 1 µM and infected with *H. pylori* SS1 at MOI of 1:100 or vehicle control (DMSO). Mixtures were incubated at 37°C for 24 hours. Quantitative PCR was performed to measure *Foxm1* mRNA levels as described above.

### *H. pylori* and thiopeptide-positive *C. acnes* coinfection in GF INS-GAS mice

The animal protocol was approved by the Massachusetts Institute of Technology Committee on Animal Care. GF INS-GAS mice on an FVB/N background [Tg(Ins1-GAS)1Sbr] were maintained in a facility accredited by AAALAC International. GF mice were housed in sterile isolators on autoclaved hardwood bedding in solid-bottomed polycarbonate cages and fed autoclaved rodent diet (Prolab RMH 3000; PMI Nutrition International, St. Louis, MO). Sterile water was provided *ad libitum*. Every other week, GF control mice isolators were confirmed negative for microbial contaminants by culture, PCR using eubacterial primers, and Gram-stained fecal smears.

Sixty-nine mice (40 males, 29 females) were included in this study. Seven- to eight-week-old male and female INS-GAS mice were infected by oral gavage with 200 µL (~2 × 10^8^ CFU) of *H. pylori* SS1 on alternate days for a total of 3 doses (*n* = 9 males, 4 females) (50). Coinfected mice were orally infected with thiopeptide-positive *C. acnes* MIT 18-1879-A3 200 µL (~2 × 10^8^ CFU) either 2 weeks prior to (*n* = 9 males, 8 females) or 1 week after *H. pylori* infection (*n* = 8 males, 6 females). Control mice were either colonized by thiopeptide-positive *C. acnes* (MIT 18-1879-A3) (*n* = 4 males, 6 females) or remained uninfected (*n* = 10 males, 5 females). Mice were necropsied at 17 weeks post-infection with *H. pylori*.

### Necropsy and histopathology

Immediately following CO_2_ euthanasia of the mice, blood was collected via cardiocentesis. Serum was separated and stored at −80°C. Gastric and mesenteric lymph nodes were collected for flow cytometry. The stomach and proximal duodenum were aseptically removed and incised along the greater curvature. Four linear gastric strips were sectioned from the lesser curvature and collected for culture, flash frozen for RNA analysis, stored at −80°C for DNA extraction, or preserved in 10% neutral buffered formalin for histopathologic evaluation (51). A comparative pathologist, who was blinded to sample identity, graded gastric lesions on an ascending scale from 0 to 4 for inflammation, epithelial defects, foveolar and glandular hyperplasia, and dysplasia and neoplasia. Scores were combined to generate a GHAI (15).

### Quantification of gastric *H. pylori*

Colonization levels of gastric *H. pylori* SS1 were quantified using qPCR in the 7500 FAST real-time PCR system (Thermo Fisher Scientific, Waltham, MA) as previously described (34). Copy numbers of *H. pylori* were normalized to micrograms of mouse chromosomal DNA in the samples using the 18S rRNA gene-based primers and probe mixture (Thermo Fisher Scientific, Waltham, MA).

### Quantitative *C. acnes* gastric colonization

Gastric tissue samples from nine *C*. *acnes*-colonized mice, four mice infected with *H. pylori* prior to *C. acnes*, and three mice dosed with *C. acnes* prior to *H. pylori* were weighed and homogenized in 1 mL of *Brucella* broth. Fifty microliters of 1:10 and 1:100 dilutions were grown in duplicate on 5% sheep blood agar plates under microaerophilic conditions. CFU counts were performed within 1 week when adequately sized colonies were present. Oral cavity swabs and feces from two animals per *C. acnes*-colonized group were cultured to confirm *C. acnes* colonization.

### Gastric FOXM1 and cytokine mRNA expression profiles in gastric samples of GF INS-GAS mice

*Il1β*, *Ifn-γ*, *Tnfα*, *Il17a*, *Il22*, *iNOS*, *Foxp3*, and *Foxm1* mRNAs were measured by quantitative PCR using commercial primers and probes for each cytokine as described previously (15).

### Cytokine protein expression in gastric samples from GF INS-GAS mice by cytokine array analysis

Gastric tissue from male mice was homogenized in liquid nitrogen with a disposable pestle (Sigma-Aldrich, St. Louis, MO). One hundred fifty microliters of lysis buffer, 500 µL of RIPA buffer with protease inhibitor (Thermo Fisher Scientific, Waltham, MA), 5-µL protease inhibitor, and 5-µL 0.5 M EDTA were added. Samples were placed in a rotating mixer at 4°C for 1 hour. Supernatant was collected following centrifugation at 10,000 × *g* for 10 minutes at 4°C. Protein concentration was measured using a BCA kit (Thermo Fisher Scientific, Waltham, MA) and adjusted to 1 mg/mL. Thirty-two-plex gastric tissue cytokine array was performed to quantify eotaxin, G-CSF, GM-CSF, IFNγ, IL-1α, IL-1β, IL-2, IL-3, IL-4, IL-5, IL-6, IL-7, IL-9, IL-10, IL-12p40, IL-12p70, IL-13, IL-15, IL-17A, IP-10, KC, LIF, LIX, MCP-1, M-CSF, MIG, MIP-1α, MIP-1β, MIP-2, RANTES, TNFα, and VEGF-A (Eve Technologies, Calgary, Alberta, Canada).

### Serology

Serum Th1-associated IgG2a and Th2-associated IgG1 antibody response to sonicated antigens of *H. pylori* and *C. acnes* were quantified by enzyme-linked immunosorbent assay (ELISA). Antigen was prepared as previously described (10). In brief, *H. pylori* SS1 and *C. acnes* were grown under microaerobic conditions for 48 hours. Bacterial cells were pelleted, washed, resuspended, and sonicated and then filtered through a 0.2-µm syringe filter to remove cell debris.

For serum IgG measurement, 96-well Immulon II plates (Thermo Fischer Scientific, Waltham, MA) were coated with antigen at a concentration of 10 µg/mL and incubated overnight at 4°C. Wells were blocked with 2% bovine serum albumin (BSA) in PBS. Serum samples were diluted 1:100 with 1% BSA in PBS. Biotinylated monoclonal anti-mouse antibodies produced by clones A85-1 (specific for mouse IgG1) and 5.7 (specific for mouse IgG2; BD Biosciences, San Jose, CA) were diluted 1:2,000 and used as secondary antibodies. Incubation with ExtrAvidin-peroxidase (1:2,000; Sigma-Aldrich, St. Louis, MO) was followed by 2,29-azinobis (3-ethylbenzthiazoline-6-sulfonic acid) diammonium salt substrate (Kirkegaard and Perry Laboratories, Gaithersburg, MD) for color development at 405/562 nm.

### Flow cytometry

GLN and MLN were manually dissociated into single cell suspensions. A total of 1–2 × 10^6^ cells were incubated with Zombie violet (BioLegend, San Diego, CA) and Fc block (anti CD16/CD32, Clone 93, BioLegend, San Diego, CA). T cell surface markers, CD4 and CD3, were detected using CD4-PerCP (1:200; Clone RM4-5; BioLegend, San Diego, CA) and CD3-APC/Cy7 (1:100; Clone 17A2; BioLegend, San Diego, CA). Cells were fixed and permeabilized using Fix/Perm solution (BioLegend, San Diego, CA). Transcription factors were detected using the following antibodies: T-bet-FITC (1:100; Clone 4B10; BioLegend, San Diego, CA), RORγT-APC (1:100; Clone B2D; Thermo Fisher Scientific, Waltham, MA), or FOXP3-PE (1:100; Clone FJK-16s; Thermo Fisher Scientific, Waltham, MA). Samples were analyzed on a BD LSR II Flow Cytometer.

### Gastric immunohistochemistry for immune cells

Paraffin-embedded gastric tissues from five control mice, four *C*. *acnes*-colonized mice, and eight mice from *H. pylori* and coinfected groups were stained using F4/80 antibody (1:100, Cell Signaling Technology, Danvers, MA) to detect macrophages, CD3 antibody (1:400, Agilent Dako, Santa Clara, CA) to detect T cells, FOXP3 antibody (1:100, Cell Signaling Technology, Danvers, MA) to detect Treg cells, CD45 B220 antibody (1:400, Thermo Scientific, Waltham, MA) to detect B cells, and MPO antibody (1:50, Thermo Scientific, Waltham, MA) to detect neutrophils by immunohistochemistry as previously described (5). Positive cells as a percentage of total cells within the gastric mucosa of whole digital slides were quantified using QuPath imaging software.

### Statistical analyses

Statistical analysis and figure creation were performed using Prism (Version 9.0, GraphPad Software, La Jolla, CA). Results shown as mean ± standard deviation. GF gastric colonization, gastric histopathology scores, *Foxm1* and cytokine mRNA expression, serology, flow cytometry % expression, and IHC results were analyzed using the Mann-Whitney U nonparametric test. Results were considered significant at *P* < 0.05 and were denoted by *P < 0.05, **P < 0.01, **P < 0.001, and ***P < 0001.

## Data Availability

Genomes for *C. acnes* strains MIT 18-1857-A1, 18-1851-A4, 18-1869-C3, and 18-1879-A3 were deposited under the following GenBank accessions, respectively: WOWG00000000, WOWH00000000, WOWI00000000, and WOWJ00000000.

## SUPPLEMENTAL MATERIAL

Supplemental material spectrum.03450-23-s0001.pdf Tables S1 and S2; Figures S1 to S6.

ASM does not own the copyrights to Supplemental Material that may be linked to, or accessed through, an article. The authors have granted ASM a non-exclusive, world-wide license to publish the Supplemental Material files. Please contact the corresponding author directly for reuse.
