# Supplementary material for: Gastric coinfection with thiopeptide-positive Cutibacterium acnes decreases FOXM1 and pro-inflammatory biomarker expression in a murine model of Helicobacter pylori-induced gastric cancer
Source: Microbiol Spectr. 2023 Nov 28;12(1):e03450-23. doi: 10.1128/spectrum.03450-23 (PMC10783005; doi:10.1128/spectrum.03450-23)
Supplement: Supplemental material — Tables S1 and S2; Figures S1 to S6. [file spectrum.03450-23-s0001.pdf]

SUPPLEMENTAL MATERIALS

Table S1. Prevalence of bacteria isolated from gastric biopsy samples from Nicaragua.

Genus	n*	Species	***Cutibacterium acnes*** **(+)**		***Cutibacterium acnes*** **(-)**	
			**HP (+)** (n=9)**	**HP (-) (n=2)**	**HP (+)** (n=15)**	**HP (-) (n=8)**
Streptococcus	25	*Streptococcus mitis*	5		8	2
		Streptococcus salivarius	4		7	2
		Streptococcus anginosus	1		1	
		Streptococcus pneumoniae	2		3	1
		Streptococcus parasanguinis	5		3	3
		Streptococcus thermophilus			2	
		Streptococcus anginosus			2	
		Streptococcus sanguinis			1	
		Streptococcus sp. (oral strain)	1		1	2
		Streptococcus cristatus			2	1
		Streptococcus infantis	3	1	1	1
		Streptococcus oralis			2	
		Streptococcus australis			2	
Rothia	19	*Rothia mucilaginosa*	5		8	2
		Rothia dentocariosa	1		3	2
Actinomyces	15	*Actinomyces odontolyticus*	2		8	3
		Actinomyces viscosus/oris			1	
		Actinomyces naeslundii				2
		Actinomyces oris			1	
		Actinomyces sp.			1	
Veillonella	7	*Veillonella dispar*	1		4	
		Veillonella parvula	1		1	

Staphylococcus	6	*Staphylococcus aureus*				1
		Staphylococcus epidermidis			2	1
		Staphylococcus pasteuri	1		1	
		Staphylococcus hominis	1			
Pseudomonas	5	*Pseudomonas aeruginosa*	2		3	
Atopobium	5	*Atopobium parvulum*	1		3	1
Prevotella	5	*Prevotella aurantiaca*			1	
		Prevotella melaninogenica			1	1
		Prevotella jejuni			1	
		Prevotella histicola	1			
		Prevotella pallens			1	
Neisseria	5	*Neisseria perflava*	1		1	
		Neisseria flavenscens			1	
		Neisseria mucosa				1
		Neisseria sicca			1	
Micrococcus	2	*Micrococcus antarcticus*		1		
		Micrococcus sp	1			
Stenotrophomonas	2	*Stenotrophomonas maltophilia*	2		1	
Elizabethkingia	2	*Elizabethkingia anophelis*	1		1	
Gemella	2	*Gemella sanguinis*			2	1
Solobacterium	2	*Solobacterium moorei*			2	
Klebsiella	1	*Klebsiella oxytoca*	1			
Enterococcus	1	*Enterococcus casseliflavus*	1			
Mogibacterium	1	*Mogibacterium diversum*	1			
Delftia	1	*Delftia acidovorans*			1	

Parvimonas	1	*Parvimonas micra*			1	
Moraxella	1	*Moraxella osloensis*			1	
Porphyromonas	1	*Porphyromonas catoniae*			1	
		Porphyromonas pasteri			1	
Corynebacterium	1	*Corynebacterium argentoratense*			1	

*n = Number of biopsies

**_H. pylori_ positivity determined by histopathology

Table S2. Other bacteria isolated from Nicaraguan gastric biopsies positive for *C. acnes*.

C. acnes **MIT strain**	***Streptococcus* spp.**	***Staphylococcus* spp.**	***Pseudomonas* spp.**	***Klebsiella* spp.**	***Rothia* spp.**	**Other Bacteria**	***Helicobacter pylori***
18-1849	*S. mitis*<br>*S. salivarius*<br>*S. anguinosus*<br>*S. pneumoniae*<br>*S. parasanguinis*	-	*P. aeruginosa*	*K. oxytoca*	*R. mucilaginosa*	*Actinomyces odontolyticus*	-
18-1851	*S. parasanguinis*<br>*S. salivarius*	-	-	-	-	-	-
18-1857	-	-	-	-	-	-	-
18-1859	*S. parasanguinis*<br>*S. salivarius*	-	-	-	*R. mucilaginosa*<br>*R. dentocariosa*	-	-
18-1863	-	-	-	-	-	-	+
18-1864	*S. infantis*<br>*S. mitis group*	-	-	-	*R. mucilaginosa*<br>*R. dentoscariosa*	*Enterococcus casseliflavus*<br>*Veillonella dispar*	+
18-1869	*S. infantis*	-	-	-	-	*Micrococcus antarcticus*	-
18-1871	*S. pneumoniae*<br>*S. salivarius*<br>*S. mitis*	*S. pasteuri*	-	-	-	*Stenotrophomas maltophilia*<br>*Micrococcus spp.*<br>*Neisseria perflava*	-
18-1873	*S. mitis*<br>*S. parasanguinis*	-	-	-	*R. mucilaginosa*	-	-
18-1879	*S. spp (oral)*	-	*P. aeruginosa*	-	-	*Stenotrophomas maltophilia*<br>*Elizabethkingia anophelis*	-
18-1881	*S. salivarius*<br>*S. infantis*<br>*S. mitis*<br>*S. parasanguinis*	*S. hominis*	-	-	*R. mucilaginosa*	*Veillonella parvula*<br>*Mogibacterium diversum*<br>*Atopobium parvulum*<br>*Actinomyces odontolyticus*<br>*Prevotella histicola*	+

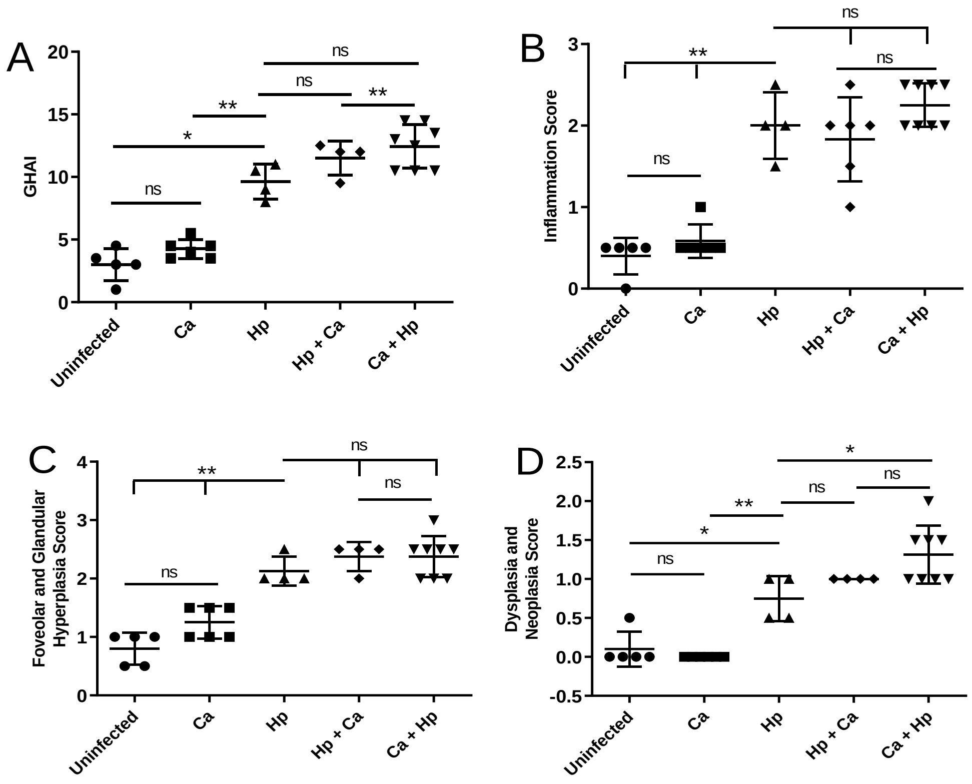

Fig S1. Gastric histopathology for female mice. (A) Combined gastric total histopathologic score (GHAI), **(B)** gastric histopathologic inflammation score, **(C)** gastric foveolar and glandular hyperplasia score, and **(D)** gastric dysplasia and neoplasia score in female mice that were uninfected, colonized by *C. acnes*, infected with *H. pylori* SS1, infected with *H. pylori* followed by *C. acnes*, or dosed with *C. acnes* prior to *H. pylori* at 17 weeks post-infection. Female mice coinfected with *C. acnes* followed by *H. pylori* showed increased dysplasia and neoplasia scores than *H. pylori* monoinfection, but no change in overall histopathology scores. Hp = *H. pylori* SS1 strain; Ca = *C. acnes*; Hp + Ca = mice infected with *H. pylori* followed by *C. acnes*; Ca + Hp = mice dosed with *C. acnes* prior to *H. pylori*. *p<0.05, **p<0.01.

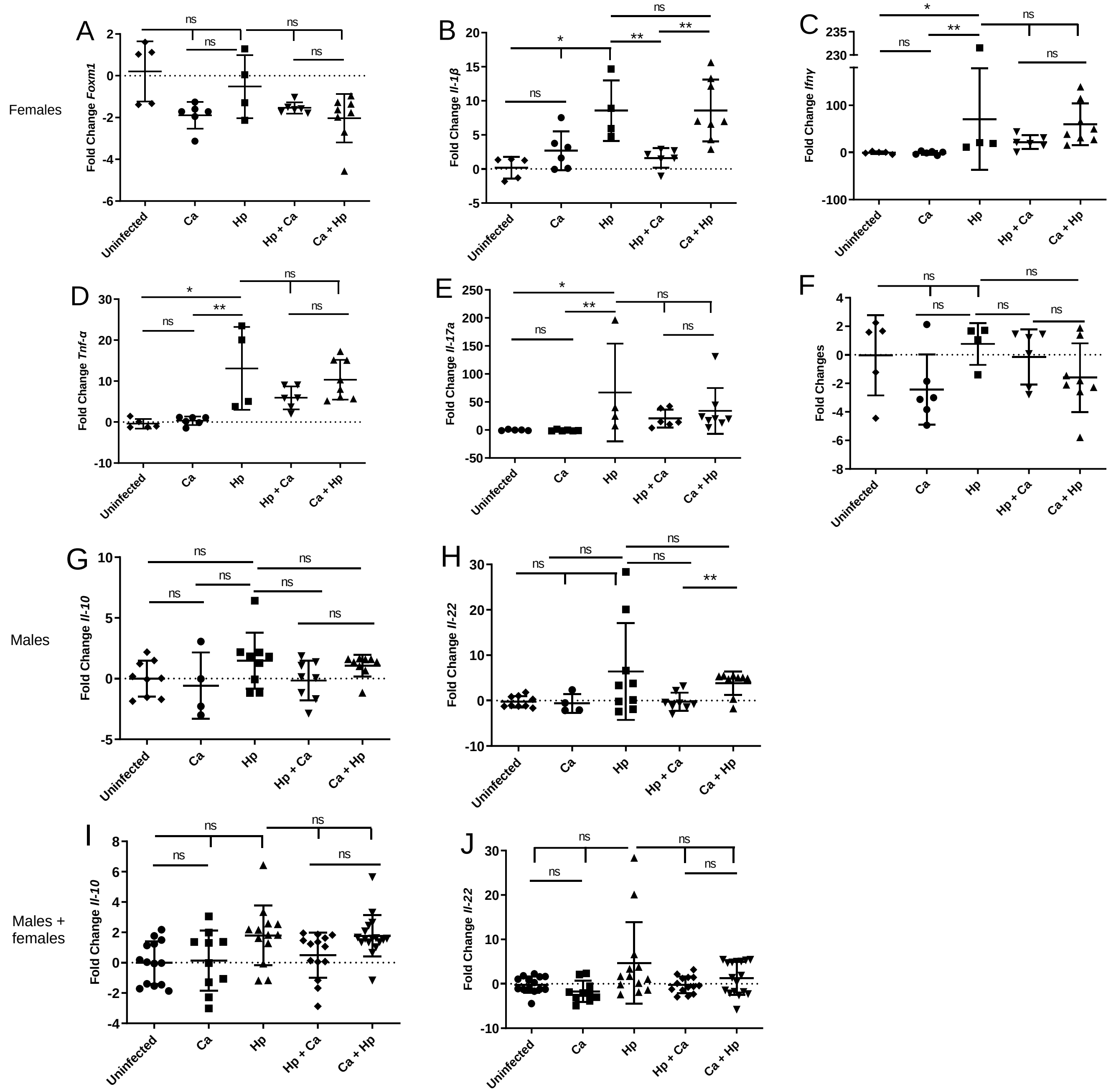

Fig S2. Cytokines and *Foxm1* gene expression in female mice. (A) Gastric mRNA levels of *Foxm1* **(A)**, *Il-1β* **(B)**, *Ifn-γ* **(C)**, *Tnf-α* **(D)**, *IL-17a* **(E)**, *Il-22* **(F)** in female mice. *Il-10* and *Il-22* mRNA expression in male mice **(G,H)** and both sexes **(I,J)** at 17 weeks post-infection. *Il-1β* expression was decreased in female mice coinfected with *H. pylori* prior to *C. acnes* compared to *H. pylori* monoinfection. Hp = *H. pylori* SS1 strain; Ca = *C. acnes*; Hp + Ca = mice infected

with *H. pylori* followed by *C. acnes*; Ca + Hp = mice dosed with *C. acnes* prior to *H. pylori*.

*$p<0.05$, **$p<0.01$.

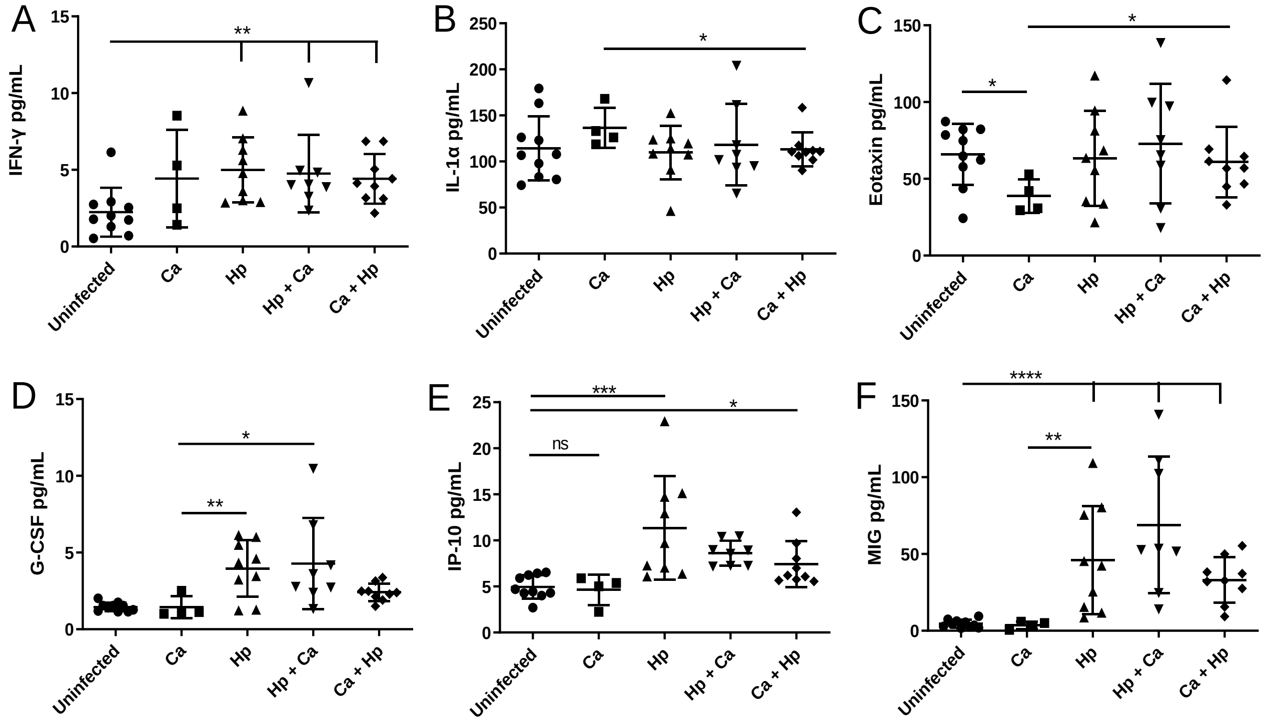

Fig S3. Gastric inflammatory proteins. (A) IFN-γ, **(B)** IL-1α, **(C)** eotaxin, **(D)** G-CSF, **(E)** IP-10, and **(F)** MIG levels in male germ-free INS-GAS mice that were uninfected, colonized by *C. acnes*, infected with *H. pylori*, infected with *H. pylori* prior to *C. acnes*, or dosed with *C. acnes* prior to *H. pylori* at 17 weeks post-infection. Inflammatory gastric proteins were increased in *H. pylori-infected* animals compared to uninfected and *C. acnes*-colonized controls. Hp = *H. pylori* SS1 strain; Ca = *C. acnes*; Hp + Ca = mice infected with *H. pylori* followed by *C. acnes*; Ca + Hp = mice dosed with *C. acnes* prior to *H. pylori*. *$p<0.05$, **$p<0.01$, ***$p<0.001$, ****$p<0.0001$.

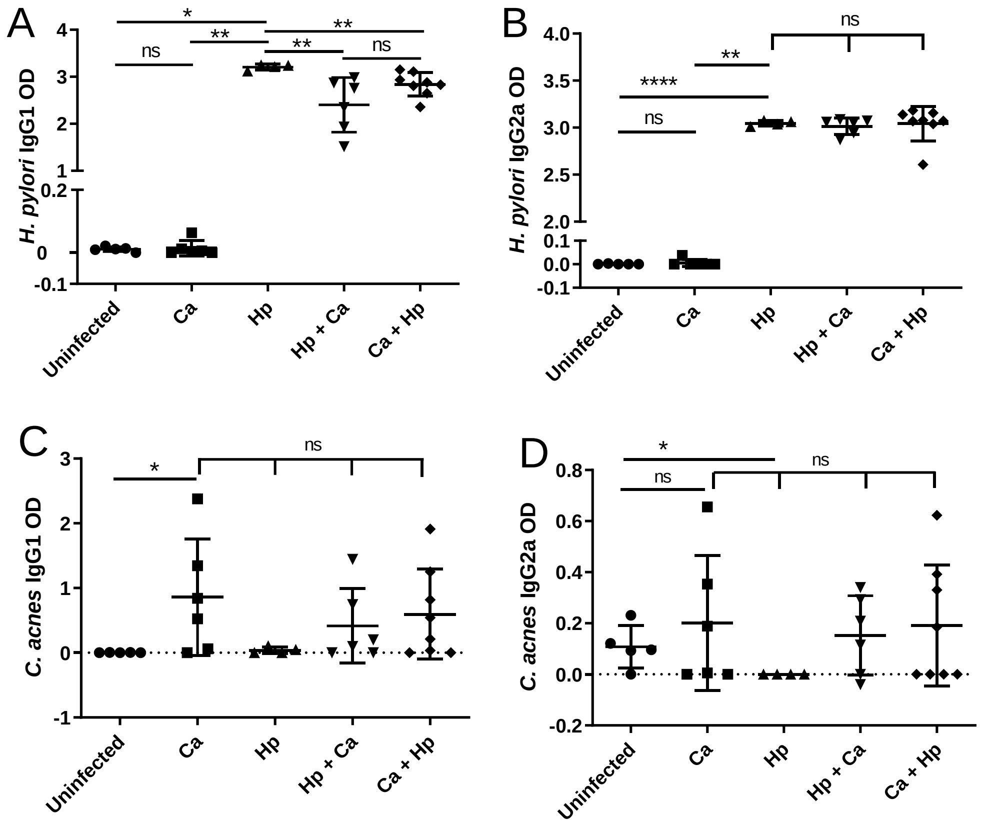

Fig S4. Antibody response against *H. pylori* and *C. acnes* in female mice. Anti-*H. pylori* and *C. acnes* serum anti-inflammatory IgG1 **(A,C)** and pro-inflammatory IgG2a **(B,D)** antibodies measured by ELISA in male germ-free INS-GAS mice that were uninfected, colonized by *C. acnes*, infected with *H. pylori*, infected with *H. pylori* prior to *C. acnes*, or dosed with *C. acnes* prior to *H. pylori* at 17 weeks post-infection. Anti-inflammatory IgG1 antibodies against *H. pylori* were decreased in coinfected females compared to *H. pylori* monoinfection. Hp = *H. pylori* SS1 strain; Ca = *C. acnes*; Hp + Ca = mice infected with *H. pylori* followed by *C. acnes*; Ca + Hp = mice dosed with *C. acnes* prior to *H. pylori*. *p<0.05, **p<0.01, ****p<0.0001.

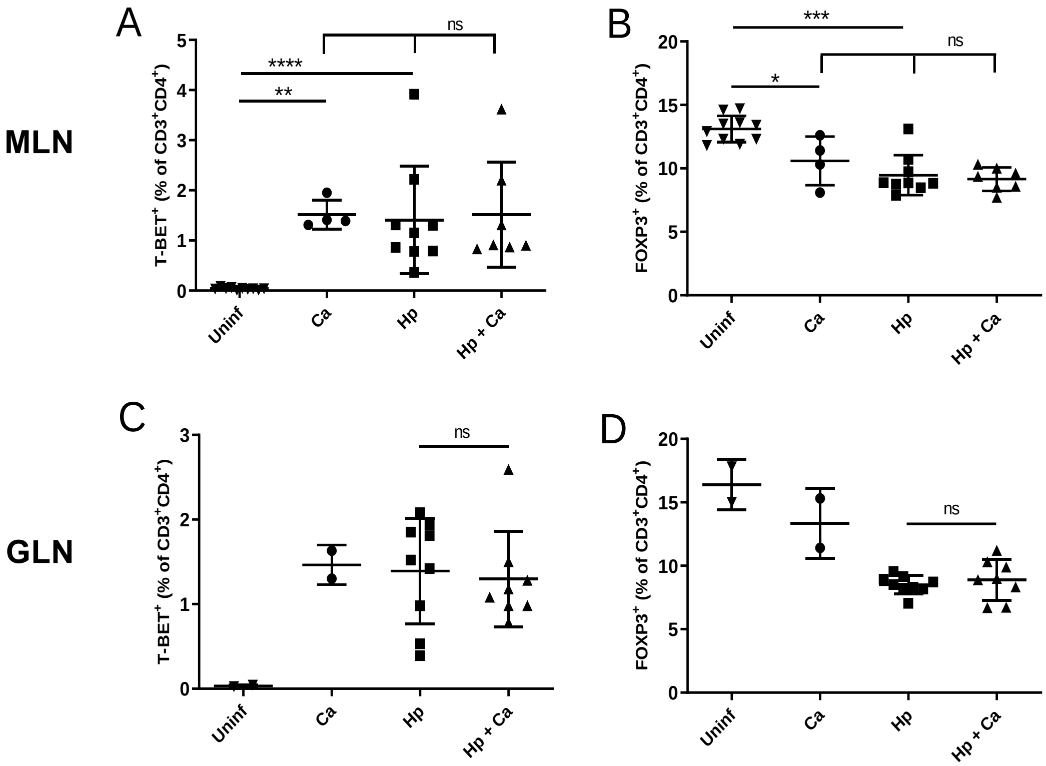

Fig S5. Flow cytometry of mesenteric and gastric lymph nodes. T-BET expression **(A)** and FOXP3 expression **(B)** in CD4+ T cells in mesenteric lymph nodes. T-BET expression **(C)** and FOXP3 expression **(D)** in CD4+ T cells in gastric lymph nodes. **(B)** FOXP3 expression in CD4+ T cells in gastric lymph nodes at 17 weeks post-infection. Colonized mice had increased T-BET and decreased FOXP3 expression compared to uninfected controls. T-BET and FOXP3 expression in gastric and mesenteric lymph nodes did not differ between coinfected and monoinfected mice. Hp = *H. pylori* SS1 strain; Ca = *C. acnes*; Hp + Ca = mice infected with *H. pylori* followed by *C. acnes*; Ca + Hp = mice dosed with *C. acnes* prior to *H. pylori*. *p<0.05, **p<0.01, ***p<0.001, ****p<0.0001.

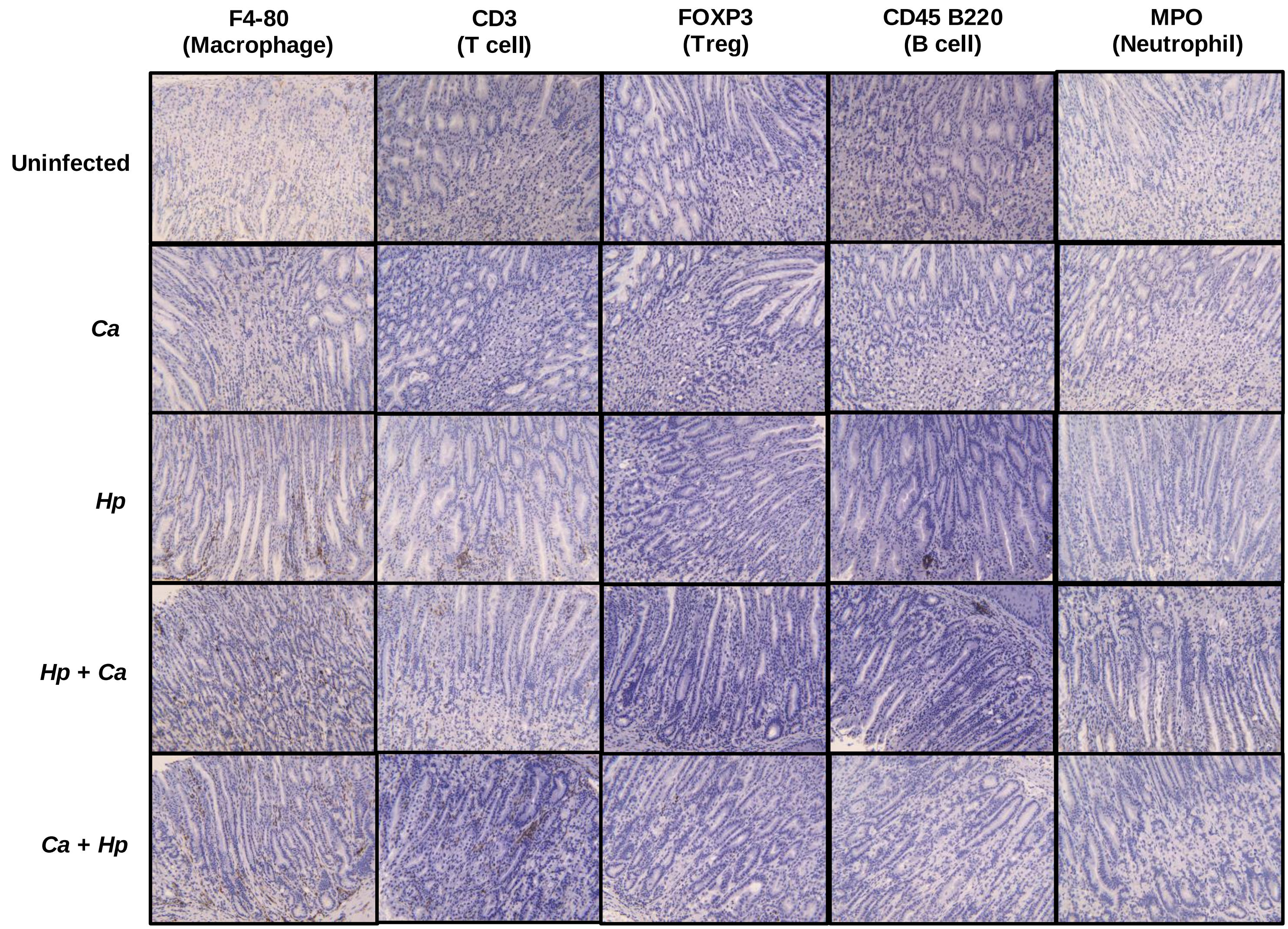

Fig S6. Representative images of gastric immunohistochemistry. Gastric samples from male mice at 17 weeks post-infection were stained for F4-80 (macrophage), CD3 (T cell), FOXP3 (Treg), CD45 B220 (B cell), and MPO (Neutrophil). Representative images were taken at 200X magnification. Gastric inflammatory cells (brown staining) were increased in *H.* pylori-infected mice compared to uninfected and *C. acnes* controls. Hp = *H. pylori* SS1 strain; Ca = *C. acnes*; Hp + Ca = mice infected with *H. pylori* followed by *C. acnes*; Ca + Hp = mice dosed with *C. acnes* prior to *H. pylori.*
